# eIF4E Enriched Extracellular Vesicles Induce Immunosuppressive Macrophages through HMGCR‐Mediated Metabolic Rewiring

**DOI:** 10.1002/advs.202506307

**Published:** 2025-08-18

**Authors:** Sonam Mittal, Minal Nenwani, Ishaque Pulikkal Kadamberi, Sudhir Kumar, Olamide Animasahun, Jasmine George, Shirng‐Wern Tsaih, Prachi Gupta, Mona Singh, Anjali Geethadevi, Chandrima Dey, Noah Meurs, Ajay Shankaran, Pradeep Chaluvally Raghavan, Deepak Nagrath, Sunila Pradeep

**Affiliations:** ^1^ Department of Obstetrics and Gynecology Medical College of Wisconsin Milwaukee WI 53226 USA; ^2^ Department of Biomedical Engineering University of Michigan Ann Arbor MI 48109 USA; ^3^ Laboratory for Systems Biology of Human Diseases University of Michigan Ann Arbor MI 48109 USA; ^4^ Department of Microbiology and Immunology Emory University School of Medicine Atlanta Georgia 30322 USA; ^5^ Department of Chemical Engineering University of Michigan Ann Arbor MI 48109 USA; ^6^ ECARI Portland Cancer Research Center Portland OR 97239 USA; ^7^ Medical College of Wisconsin Cancer Center Medical College of Wisconsin Milwaukee WI 53226 USA; ^8^ Department of Physiology Medical College of Wisconsin Milwaukee WI 53226 USA; ^9^ Rogel Cancer Center University of Michigan Ann Arbor MI 48106 USA

**Keywords:** eIF4E, extracellular vesicles, metabolism, ovarian cancer, PD‐L1, tumor associated macrophages

## Abstract

Tumor driven immune suppression poses a significant impediment to the success of immunotherapy in ovarian cancer. Among the various mechanisms contributing to immune suppression, intracellular communication facilitated by tumor‐derived extracellular vesicles (EVs) within the tumor microenvironment emerges as a pivotal factor influencing tumor growth. Here, it is demonstrated that extracellular vesicle‐packaged eIF4E from tumor cells alters protein translation in macrophages, contributing to antitumor immune response. Mechanistically, tumor derived EV‐packaged eIF4E significantly enhances the expression of 3‐hydroxy‐3‐methyl‐glutaryl‐coenzyme A reductase (HMGCR), driving the synthesis and secretion of cholesterol. This, in turn, activates macrophages and causes immunosuppression through the X‐box binding protein 1 and Programmed death‐ligand 1 (XBP1/PD‐L1) axis. Strikingly, both genetic and pharmacological depletion of HMGCR in macrophages effectively restores their antitumor activity. Clinically, elevated HMGCR expression in tumor‐associated macrophages is associated with poor survival outcomes in ovarian cancer patients. The pivotal role of eIF4E is underscored here as a key signaling mediator, facilitating the communication between tumor and immune cells via EVs to promote immune suppression and suggesting HMGCR as a potential therapeutic target for tumor immunotherapy.

## Graphical abstract

Here, it is revealed that ovarian cancer cells release extracellular vesicles enriched with eIF4E, which alters protein production in macrophages. This leads to increased expression of 3‐hydroxy‐3‐methyl‐glutaryl‐coenzyme A reductase (HMGCR), boosting cholesterol synthesis and activating an immunosuppressive pathway through X‐box binding protein 1 and Programmed death‐ligand 1 (XBP1/PD‐L1). High HMGCR levels in tumor‐associated macrophages are linked to poor survival in ovarian cancer, identifying HMGCR as a potential therapeutic target to overcome tumor‐induced immune suppression.

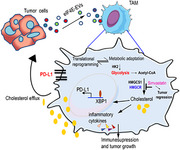

## 1 Introduction

Effective communication between cancer cells and surrounding cells within the tumor microenvironment (TME) is a critical factor influencing tumor progression. There has been a growing emphasis on understanding the role of tumor‐derived extracellular vesicles (EVs) in orchestrating the intricate intracellular communications within the TME.^[^ ^1^, ^2^, ^3^ ^]^ However, the mechanisms of EVs crosstalk in TME for oncogenic actions are not well understood. EVs, ranging from 50 to 300 nm, carry multiple specific proteins or RNAs secreted by host cells.^[^ ^4^ ^]^ These molecules precisely target recipient cells, influencing tumor behavior and angiogenesis.^[^ ^5^, ^6^ ^]^ Our recent work has reported that tumor‐derived EVs cause T‐cell exhaustion in the TME through sphingosine‐mediated signaling and impacting immunotherapy outcomes in ovarian cancer.^[^ ^2^ ^]^ Emerging evidence suggests that the molecular information contained in EVs can be harnessed for diagnostic and therapeutic purposes.^[^ ^7^, ^8^, ^9^, ^10^, ^11^ ^]^

The levels of the individual components of the eIF4F complex, and its activity, are elevated in many human cancers, constituting vulnerability for transformed cells.^[^ ^12^ ^]^ The initial recognition of the messenger RNA (mRNA) 5′‐end involves the translation factor eIF4F, a heterotrimeric complex formed by the assembly of eIF4A, eIF4E, and eIF4G. eIF4E is the cap‐binding protein that specifically binds to the 5′‐cap structure. eIF4E also interacts with the scaffold protein eIF4G. eIF4G, in turn, binds to eIF4A, a DEAD‐box helicase that has been implicated in the unwinding of double‐stranded regions in the 5′ untranslated region (5′‐UTR) that might otherwise interfere with protein initiation complx (PIC) recruitment and scanning.^[^ ^13^ ^]^ Early studies demonstrated that eIF4E overexpression enhances the translation of mRNAs with structural repeats in the 5′UTR. The average well‐translated mRNA has a 5′UTR of 20–50 nucleotides. Around 10% of cellular mRNAs contain atypically long 5′UTR and many of these encode proto‐oncogenes, antiapoptotic proteins, and growth factors. A long 5′UTR and Guanine‐Cytosine (GC) rich sequence tends to form a stable secondary structure. It has been shown that the translation of mRNAs with long 5′UTRs is often sensitive to the expression levels of eIF4E and eIF4A1. While it is established that endogenous levels of eIF4F complex proteins are elevated in the TME, there is limited reporting on their encapsulation within EVs. Our data show that the presence of eIF4E in ovarian cancer derived EVs underscores its functional significance in these vesicles. This encapsulation of eIF4E within EVs holds significant implications as it can facilitate the transfer of eIF4E into other cells in the TME, thereby augmenting tumorigenic potential.

The TME comprises a dynamic cellular environment surrounding the tumors, encompassing macrophages, stroma, stem cells, fibroblasts, lymphocytes, pericytes, adipocytes, and blood vessels.^[^ ^14^ ^]^ Tumor‐associated macrophages (TAMs) represent the majority of immune cells found in ascites, making them the predominant immune cell type in ovarian cancer.^[^ ^15^ ^]^ Tumor cells released EVs influence macrophages, modulating immunity, regulating inflammation, and impacting the TME.^[^ ^16^ ^]^ Therefore, understanding macrophage dynamics in ovarian cancer ascites is crucial for developing targeted therapeutic approaches to disrupt the tumor promoting environment and improve treatment outcomes.

Our data demonstrate that tumor‐derived EVs, through the action of eIF4E, impact the protein synthesis in macrophages, notably augmenting the translation of genes associated with cellular metabolism. Furthermore, our findings elucidate that eIF4E‐EVs promote tumor formation by inducing the accumulation of cholesterol in macrophages via 3‐hydroxy‐3‐methyl‐glutaryl‐coenzyme A reductase (HMGCR), resulting in an immunosuppressive phenotype in the TME. Therefore, inhibiting HMGCR in macrophages or reducing cholesterol levels within the TME has the potential to suppress tumor growth. Our research sheds light on the mechanism whereby tumor cells exploit EVs to transport eIF4E, a critical translation‐regulating molecule, to macrophages, thereby dampening antitumor immune responses.

## 2 Results

### 2.1 Tumor Derived EVs Enriched in Translational Regulators eIF4E and eIF4A1 Drive Ovarian Cancer Progression

Several studies have shown that ovarian cancer cells release large quantities of EVs into patient ascites and plasma. These EVs, specifically derived from tumor cells, are anticipated to carry a molecular signature that partly mirrors that of the parental tumor cells.^[^ ^17^ ^]^ Furthermore, these EVs are enriched in molecules known to promote tumor growth.^[^ ^17^, ^18^ ^]^ A comprehensive proteomic analysis was conducted to investigate the impact of EVs derived from ovarian cancer cells on tumor growth and metastasis. EVs were isolated from ovarian cancer cell lines and characterized using nanoparticle tracking analysis (NTA), which confirmed their size distribution within the expected range of 50–300 nm. (Figure S1A–D, Supporting Information). Transmission electron microscopy (TEM) further validated their size and morphology, which was consistent with the standards established in the Minimal Information for Studies of Extracellular Vesicles (MISEV) guidelines (Figure S1E, Supporting Information). Additionally, the presence of EVs in our preparations was validated by the detection of proteins typically enriched in EVs (ALIX, TSG101, CD63) and the exclusion of intracellular proteins that are not shed in EVs (GM130) by western blot (Figure S1F, Supporting Information).

**Figure 1:**
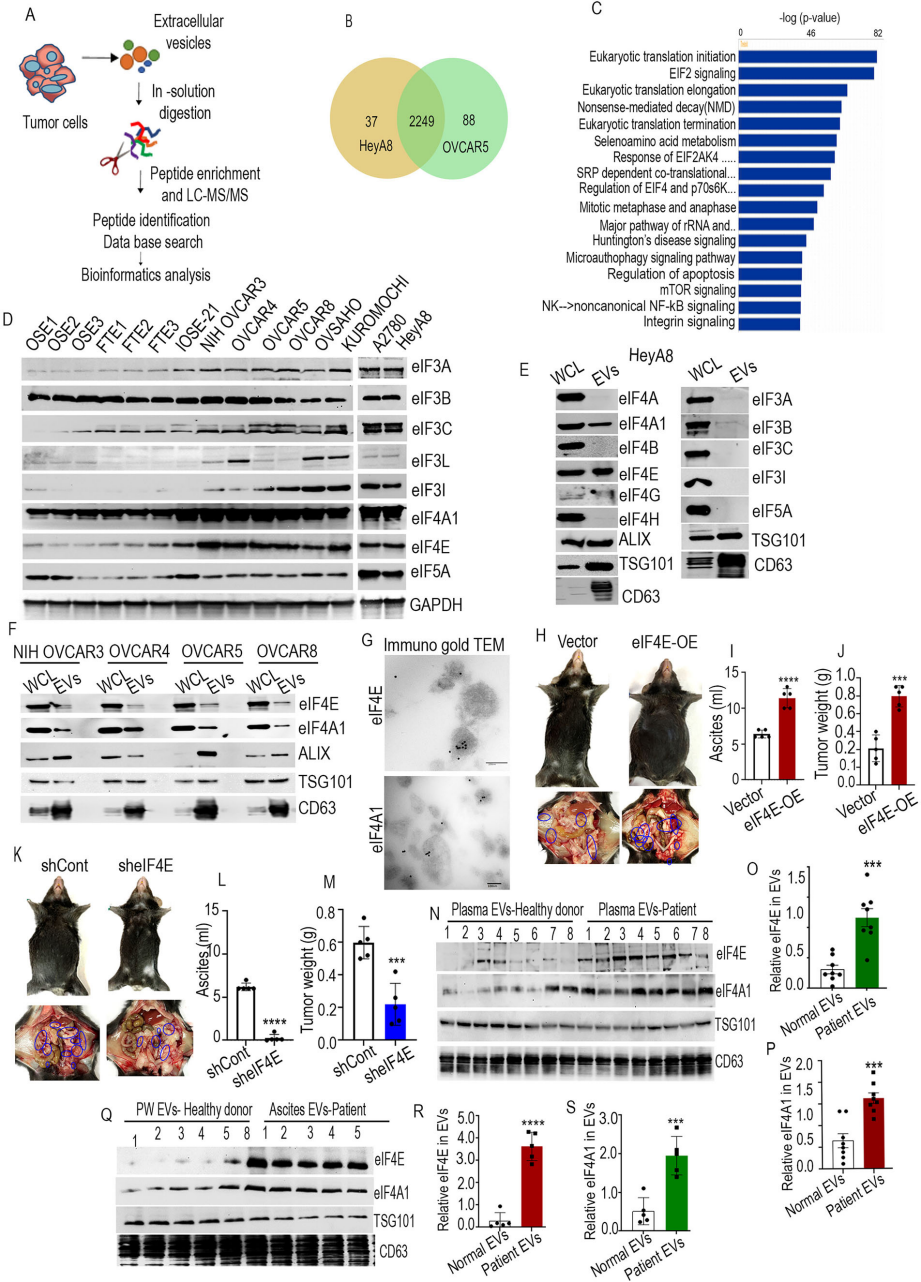
Extracellular vesicle isolation and proteomic analysis. A) EV isolation and proteomics workflow. B) Venn diagram of total proteins identified in HeyA8 and OVCAR5 cell derived EVs by LC‐MS/MS. C) Pathway analysis for proteins identified in HeyA8 and OVCAR5 cell derived EVs using Ingenuity Pathway Analysis (IPA). The top pathways were ranked based on *p*‐value and the bars represent the inverse log of the *p*‐value (*x*‐axis). D) Expression of the most abundant eukaryotic translation initiation proteins identified. E,F) Western blot analysis for different eIFs proteins on isolated EVs and whole cell lysates. G) Representative TEM image of OVCAR8 cells derived EVs. EVs were immunogold‐labeled with anti‐eIF4E and anti‐eIF4A1 antibodies. Scale bars: 200 nm (upper image) and 100 nm (lower image). H) Representative image of enlarged peritoneal cavity and increased tumor burden (blue circles) in mice with eIF4E overexpressed ID8 *Trp53^−/−^;Brca2^−/−^* cells (*n* = 5). I) Bar graph representing the ascites volume and tumor weight of mice from the vector control and eIF4E overexpressed groups. K) Representative images of the peritoneal cavities and tumor locations (blue circles) of mice from the sh‐control and sheIF4E groups (*n* = 5). L) Bar graphs representing ascites volume and M) tumor weight. N) Validation of different proteins in EVs isolated from the plasma of healthy donors and ovarian cancer patients (*n* = 8). O,P) Densitometry analysis of the experiment, as in (N). Q) Validation of different proteins in EVs isolated from the pelvic wash of healthy donors and ascites of ovarian cancer patients (*n* = 5). R,S) Densitometry analysis of the experiment, as in (Q). Error bars indicate mean ± standard error of the mean (SEM) Significance was determined by Student's *t*‐test, where ∗∗∗*p* < 0.001, ∗∗∗∗*p* < 0.0001.

Mass spectrometric analysis of EVs isolated from HeyA8 and OVCAR5 cells, using liquid chromatography‐tandem mass spectrometry (LC‐MS/MS), identified a total of 2249 proteins common to the EVs from both cell lines (**Figure** **1**A,B and Table S1, Supporting Information). The results from Ingenuity Pathway Analysis (IPA) underscored proteins that were highly enriched in pathways related to eukaryotic translational regulation (Figure 1C). HeyA8 and OVCAR5 cells represent different molecular subtypes and exhibit varying degrees of aggressiveness and drug responsiveness, thereby capturing the heterogeneity of high‐grade serous ovarian cancer. By analyzing the common EV‐associated proteins from these divergent cell lines, we aim to identify conserved cargo elements that may be central to EV‐mediated communication in ovarian cancer.

To narrow down our search for a specific translational regulator, we evaluated the protein expression within these pathways across various ovarian cancer cell types. Among the translational regulators enriched in EVs from HeyA8 and OVCAR5 cells, the eIF3, eIF4, and eIF5A proteins stood out as the most abundant. Immunoblotting confirmed that these proteins were higher in ovarian cancer cells compared to primary ovarian surface epithelial (OSE) and fallopian tube surface epithelial cells (Figure 1D). We sought to determine the presence of eIF3, eIF4, and eIF5A factors in EVs isolated from HeyA8 cells to validate the mass spectrometry data. Immunoblotting confirmed the presence of eIF4E and eIF4A1 in both whole cell lysate and EVs isolated from the supernatants of HeyA8 cells. However, a little expression of eIF4G was reported in EVs from HeyA8 cells. (Figure 1E). eIF4E and eIF4A1 were also present in EVs isolated from multiple human and murine ovarian cancer cell lines (Figure 1F and Figure S1G, Supporting Information). eIF4E and eIF4A1 were not detected in EVs isolated from normal ovarian epithelial cells, including OSE and immortalized ovarian surface epithelial‐21 (IOSE‐21) cells (Figure S1H,I, Supporting Information). TEM images using immunogold staining for eIF4E and eIF4A1 confirmed the presence of these proteins within tumor cells derived EVs (Figure 1G). We also compared our proteomic data with the ExoCarta database and identified the presence of both eIF4E and eIF4A1 in EVs derived from ovarian cancer cells.^[^ ^19^, ^20^ ^]^

To ensure the purity of the EV preparations enriched in eIF4E and eIF4A, sucrose gradient centrifugation was employed. This is a widely recognized EV fractionation technique that separates EVs from soluble proteins and nucleic acids.^[^ ^21^ ^]^ In line with established EV fractionation protocols, fractions 3–5 (of 8), representing the 20–40% sucrose gradient, exhibited the highest levels of EV markers (CD63 and ALIX), along with eIF4E and eIF4A1 (Figure S1J, Supporting Information); consequently, these fractions were selected for all our future experiments.

To confirm the notion that eIF4E and eIF4A1 are indeed transported by EVs, EV release from tumor cells was selectively inhibited using dimethyl amiloride (DMA) (20 µg mL^−1^). DMA inhibits EV biogenesis primarily by targeting the endosomal sorting and trafficking machinery, particularly pathways involved in exosome formation and release.^[^ ^22^ ^]^ DMA significantly reduced the level of eIF4E and eIF4A1 in EVs, suggesting that blocking EV biogenesis inhibits the packaging of these proteins inside EVs without affecting the intercellular levels (Figure S1K,L, Supporting Information).

To elucidate the distinct functions of eIF4E and eIFA1, EVs enriched or depleted in these proteins were generated by altering the expression levels of eIF4E, eIFA1, and other EV constituents. In vivo data demonstrated that C57BL/6 female mice implanted with ID8 *Trp53^−/−^;Brca2^−/−^* murine ovarian cancer cell lines overexpressing eIF4E or eIF4A1 exhibited an increased volume of ascites and tumor burden within the peritoneal cavity compared to the control group (Figure 1H–J and Figure S1M–P, Supporting Information). Similarly, mice implanted with eIF4E and eIF4A1 knockdown (KD) ID8 *Trp53^−/−^;Brca2^−/−^* cells (Figure S1Q,R, Supporting Information) had a decreased volume of ascites and tumor burden as compared to the control group (Figure 1–M and Figure S1S–U, Supporting Information). Immunohistochemistry (IHC) and western blotting analysis also revealed a significant increase in eIF4E and Ki67 levels in tumor tissue after eIF4E overexpression (Figure S1V, Supporting Information) and a significant decrease in eIF4E and Ki67 levels in the eIF4E KD group (Figure S1W, Supporting Information).

Moreover, a significant increase in the expression of eIF4E and eIF4A1 in EVs from the plasma and ascites of ovarian cancer patients was observed, compared to EVs from the plasma and pelvic wash of healthy donors. (Figure 1N–S).

### 2.2 TSG101‐Mediated Recruitment of eIF4E and eIF4A1 into EVs Enhances Protein Synthesis in Macrophages

**Figure 2:**
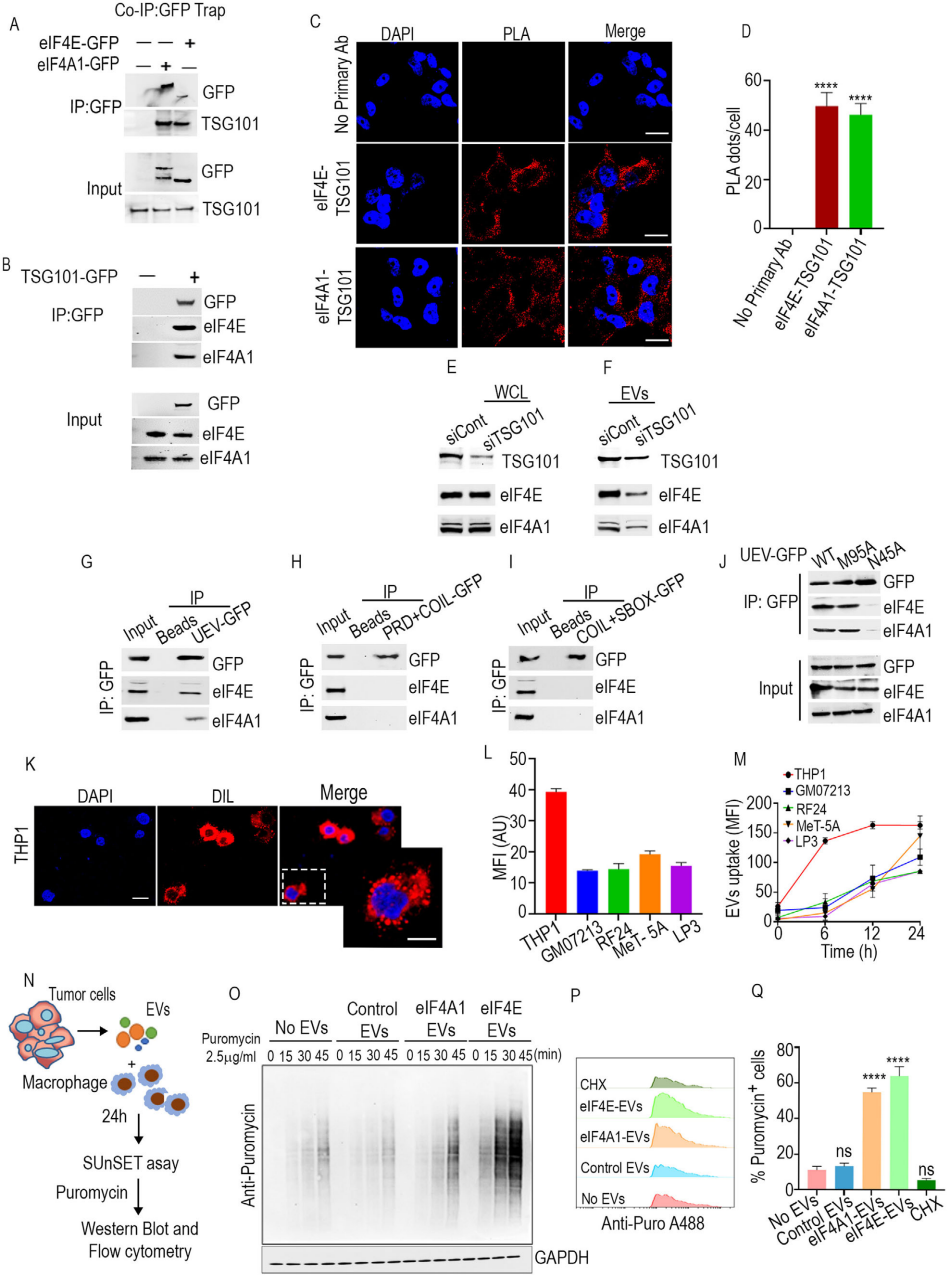
Translation initiation factors in EVs enhance protein synthesis in macrophages. A) Immunoprecipitation of eIF4E‐GFP and eIF4A1‐GFP fusion proteins with their binding partner TSG101 using GFP‐tag beads. B) Reverse immunoprecipitation of TSG101‐GFP fusion protein and its interacting partners, eIF4E and eIF4A1. C) In‐situ proximity ligation assay (PLA). Red dots represent the interaction of eIF4E and eIF4A1 with TSG101. Nuclei were stained with 4′,6‐diamidino‐2‐phenylindole (DAPI) (blue). Scale bar 50 µm. D) Bar graph representing the mean ± SEM of red PLA dots counted using Image J software. E,F) Immunoblot analysis of the whole cell lysate (WCL) and EVs fraction after TSG101 knockdown in OVCAR8 cells. G) Co‐IP of TSG101‐UEV‐GFP, eIF4E, and eIF4A1. H) Co‐IP of TSG101‐PRD+COIL‐GFP, eIF4E, and eIF4A1. I) Co‐IP of TSG101‐COIL+SBOX‐GFP, eIF4E, and eIF4A1. J) Western blotting of Co‐IP assay of TSG101‐UEV wildtype (WT) and mutants (M95A, N45A) with eIF4E and eIF4A1. K) DIL‐labeled EV expression in THP1‐derived macrophages. Scale bars 50 and 20 µm. L) Bar graph showing the mean fluorescence intensity (MFI) of DIL‐labeled EVs in THP1‐derived macrophages, GM07213 fibroblasts, RF24 endothelial, and MeT5A and LP3 mesothelial cells. M) Kinetics of DIL‐labeled EVs uptake in THP1‐derived macrophages, GM07213 fibroblasts, RF24 endothelial, and MeT5A and LP3 mesothelial cells. N) Schema illustrating the principle of surface sensing of translation (SUnSET) assay. O) SUnSET measurements of protein synthesis by western blotting in THP1‐derived macrophages. P,Q) FACS of the puromycin‐labeled cells using the antipuromycin antibody tagged with Alexa Fluor‐488. Error bars indicate mean ± SEM. Significance was determined by Student's *t*‐test and one‐way analysis of variance (ANOVA), where ****p < 0.0001. ns, nonsignificant.

Proteins associated with the endosomal sorting complex required for transport (ESCRT), such as TSG101, are responsible for recruiting cargo proteins into EVs.^[^ ^23^ ^]^ A green fluorescent protein (GFP)‐trap co‐immunoprecipitation assay was used to understand whether eIF4E and eIF4A1 are packaged into EVs by directly interacting with ESCRT proteins. GFP‐tagged beads were employed to precipitate eIF4E‐GFP and eIF4A1‐GFP from the lysates of OVCAR8 cells, which stably express eIF4E and eIF4A1 GFP fusion proteins (**Figures** **2**A and S2A,B, Supporting Information). This assay revealed a unique interaction between the ESCRT‐I component, TSG101, and both eIF4E and eIF4A1. To confirm these interactions, reciprocal immunoprecipitation was performed using OVCAR8 cells expressing TSG101‐GFP. The results showed that TSG101 co‐immunoprecipitated with eIF4A1 and eIF4E, confirming its interaction with these proteins and their encapsulation into EVs (Figure 2B).

To further investigate the interaction between eIF4E and eIF4A1 with TSG101, a proximity ligation assay (PLA) was employed to detect proximity between eIF4E‐TSG101 and eIF4A1‐TSG101. There was a significant increase in the PLA intensity when primary antibodies against the two binding partners, either eIF4E or eIF4A1 and TSG101, were used, indicating the physical closeness between both eIF4E‐TSG101 and eIF4A1‐TSG101 (Figure 2C,D).

Next, we sought to determine whether TSG101 plays a role in recruiting eIF4E and eIF4A1 into EVs. To accomplish this, TSG101 was depleted using siRNA in OVCAR8 cells. EVs were then isolated from the supernatant and analyzed for eIF4E and eIF4A1 content. Western blotting revealed a significant reduction in the levels of eIF4E and eIF4A1 in EVs isolated from TSG101 knockdown cells, but not in the whole cell lysate, indicating the involvement of TSG101 in recruiting eIF4E and eIF4A1 into EVs (Figure 2E,F).

Our subsequent objective was to identify the TSG101 domain essential for its binding to eIF4E and eIF4A1, thereby facilitating their sorting into EVs. Previous studies have established that the ubiquitin‐E2‐like variant (UEV) domain of TSG101 protein interacts with the ubiquitin moiety of proteins designated for sorting or degradation.^[^ ^24^, ^25^, ^26^ ^]^ For instance, TSG101 recognizes ubiquitin through the UEV domain, which is required for sorting epidermal growth factor receptor  (EGFR) into multivesicular bodies (MVBs).^[^ ^25^, ^27^ ^]^ To address this, co‐immunoprecipitations (Co‐Ips) were conducted with three truncated TSG101 proteins (Figure S2C–E, Supporting Information). Our results revealed that only the UEV domain directly interacts with eIF4E and eIF4A1 (Figure 2G–I). Further experiments showed that mutations in TSG101 UEV domain amino acids known to be crucial for TSG101 interaction with ubiquitin (N45A), abolished the interaction of TSG101 with eIF4E and eIF4A1, whereas the replacement of other amino acids in the UEV domain (M95A) of TSG101 had no distinct effect (Figure 2J). Taken together, our data provide compelling evidence that the UEV domain is important for the binding of TSG101 to eIF4E and eIF4A1, thus for facilitating their encapsulation into EVs.

Following this, the uptake efficacy of tumor cells derived EVs by various cell types was determined. The EVs were isolated from OVCAR8 cells and fluorescently labeled with 1,1′‐dioctadecyl‐3,3,3′,3′‐tetramethylindocarbocyanine perchlorate (DIL) dye. These labeled EVs were subsequently incubated with THP1‐derived macrophages, fibroblasts (GM07213), endothelial (RF24), and mesothelial (MeT5A, LP3) cells (Figure 2K,L and Figure S2F, Supporting Information). Our observations revealed that macrophages exhibited increased internalization of EVs compared to the other cell types. Furthermore, we delved into the dynamics of internalization. Consistent with the above observations, the temporal patterns of EV internalization exhibited two distinct patterns. In the case of macrophages, the uptake of EVs significantly increased over time, reaching a plateau after 6 h. Conversely, for other cell types, efficient detection of EV uptake was observed at 24 h of incubation (Figure 2M and Figure S2G, Supporting Information).

To elucidate the impact of EV‐packaged eIF4E/eIF4A1 on global translational regulation, macrophages, endothelial cells, and fibroblast cells were incubated for 24 h with EVs isolated from (I) parental OVCAR8 and OVCAR5 cells and (II) OVCAR8 and OVCAR5 cells that overexpress eIF4A1 or eIF4E (Figures S2A,B and S2H–J, Supporting Information). Total protein synthesis was assessed using the Surface Sensing of Translation (SUnSET) assay, which employs an antipuromycin antibody for immunological detection.^[^ ^25^ ^]^ During the newly synthesized polypeptide chain, puromycin is incorporated into the nascent polypeptide chain, thereby inhibiting the formation of a new peptide bond with the subsequent aminoacyl‐tRNA and terminating peptide elongation (Figure 2N). This leads to the release of a truncated, puromycin‐bound peptide from the ribosome. Two independent experiments were conducted based on the SUnSET assay principle. First, immunoblotting was performed using an antipuromycin antibody. In a separate experiment, immunostaining was performed with a fluorochrome‐labeled antipuromycin antibody, followed by flow cytometry analysis. Both assays demonstrated an increase in protein synthesis rates, notably in macrophages compared to the other cell types (Figures 2O–Q and S2K–R, Supporting Information). These findings prompted us to hypothesize that macrophages undergo significant reprogramming when exposed to EVs released by ovarian cancer cells within the tumor microenvironment.

### 2.3 EV‐Packaged eIF4E Induces Metabolic Reprogramming in Macrophages

**Figure 3:**
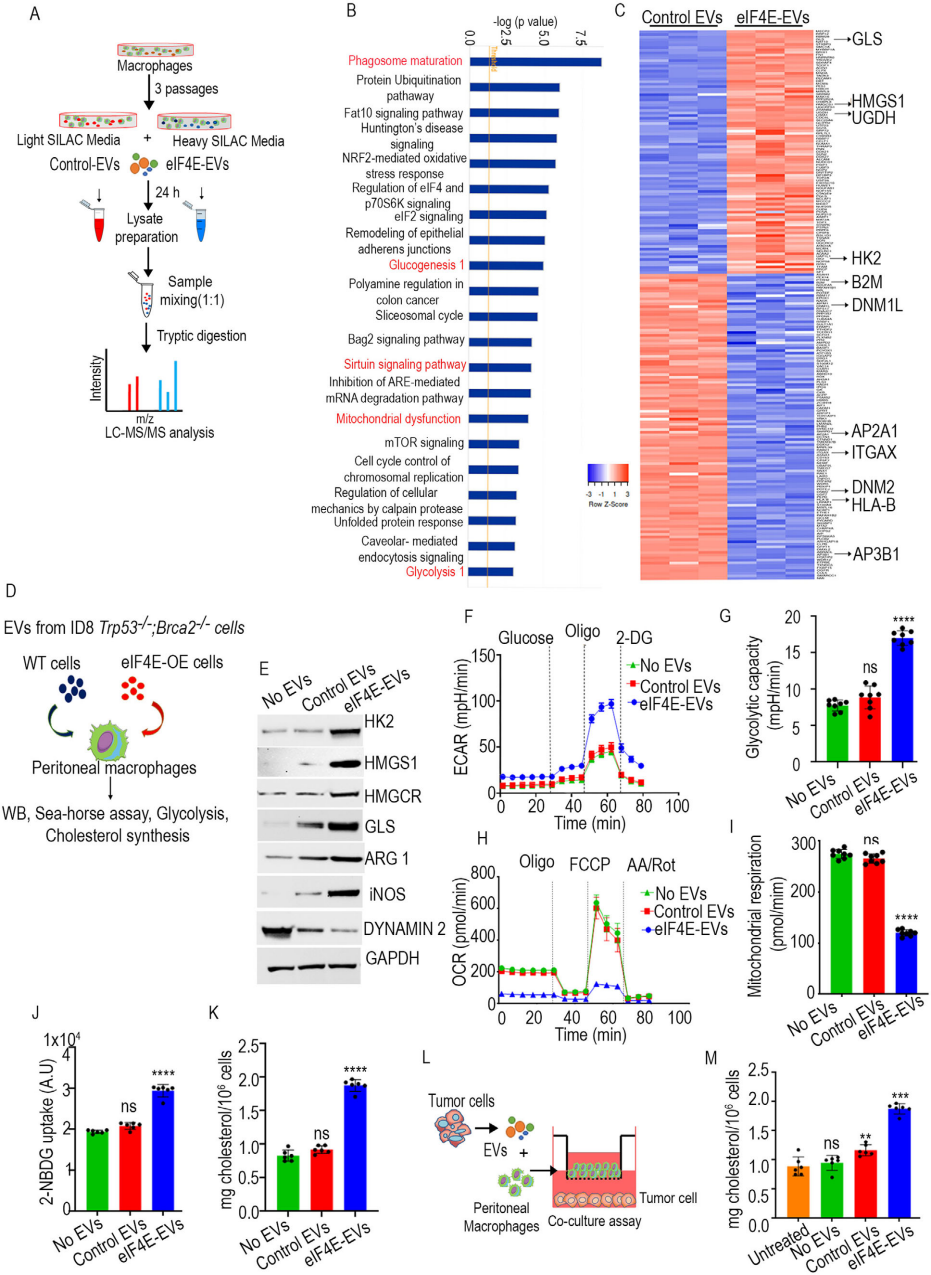
eIF4E‐loaded EVs induce metabolic reprogramming in macrophages. A) Schema representing SILAC‐MS workflow. B) IPA analysis of signaling pathways identified in THP1‐derived macrophages treated with EVs. C) Heat map showing significantly upregulated and downregulated proteins. D) Workflow representing validation of SILAC data. E) Representative western blots showing expression of HK2, HMGCS1, HMGCR, GLS, ARG1, iNOS, and DYNAMIN 2 in peritoneal macrophages treated with EVs. F,G) Seahorse glycolysis stress test with sequential addition of glucose, oligomycin, and 2‐deoxy‐ d‐glucose (2‐DG) in peritoneal macrophages pretreated with eIF4E‐EVs or control EVs. Data are representative of three independent experiments. H,I) OCR measurement in tumor derived EVs‐stimulated macrophages (*n* = 3). J) Glucose uptake assay with 2‐NBDG in macrophages stimulated with EVs (*n* = 3). K) Cholesterol synthesis in macrophages stimulated with eIF4A1‐EVs by Amplex cholesterol assay (*n* = 3). L) Schema of coculture assay. M) Cholesterol measurement in ID8 *Trp53^−/−^;Brca2^−/−^* cells cocultured with peritoneal macrophages treated with EVs (*n* = 3). Error bars indicate mean ± SEM, *p < 0.05, **p < 0.01, ***p < 0.001, ****p < 0.0001 (one‐way ANOVA). ns, nonsignificant.

To gain a comprehensive understanding of the most affected biological processes and the proteins that are either upregulated or downregulated by eIF4A1 and eIF4E loaded EVs, stable isotope labeling by amino acids in cell culture (SILAC) proteomic analysis in cultured THP1‐derived macrophages was performed (**Figure** **3**A). Macrophages were treated with EVs derived from OVCAR8 cells, and mass spectrometry was performed. In this analysis, a total of 1639 proteins were identified using the SILAC‐MS in THP1‐derived macrophages. Among these, 235 proteins exhibited significant upregulation, while 458 proteins showed significant downregulation in response to eIF4E‐EVs compared to control EVs (Table S2, Supporting Information). Gene Ontology (GO) analysis indicated that the pathways related to phagosome maturation and metabolic alterations were specifically enriched in the eIF4E‐EV treated group (Figure 3B). In contrast, treatment with eIF4A1‐derived EVs did not result in the enrichment of pathways related to metabolic processes, indicating a distinct functional divergence and potential cellular effects of eIF4E‐EVs versus eIF4A1‐EVs. This could be because the availability and/or activity of individual eIF4F components has been shown to have a selective impact on the translation of specific mRNAs, which needs to be further analyzed. Although the mechanisms underlying this selectivity are incompletely understood, features within the 5′ UTR appear to be the main determinant. For instance, the translation of mRNAs with very short 5′ UTRs is disproportionally reduced, compared with global translation rates, upon inhibition of eIF4E activity, while remaining relatively insensitive to the perturbation of eIF4A activity. Subsequently, the proteins with a ±1.2‐fold difference associated with these pathways in our SILAC dataset were identified, and a heat map was generated. Proteins associated with key metabolic pathways such as glucose (HK2), glutamine (GLS), and cholesterol metabolism (HMGCS1, LIMA1) were upregulated, while proteins involved in phagocytosis and endocytosis (DNM1L, DNM2, AP2A1, and AP3B1) displayed downregulation (Figure 3C).

The SILAC data validation involved western blot analysis on peritoneal macrophages isolated from naïve mice treated with EVs from WT and eIF4E overexpressing cells. eIF4E‐EVs induced increased expression of HK2, GLS, HMGCS1, and HMGCR. Both HMGCS1 and HMGCR are the rate‐limiting enzymes in the mevalonate pathway for cholesterol synthesis.^[^ ^28^ ^]^ Additionally, eIF4E‐EV stimulation led to increased expression of iNOS and arginase 1 (ARG1) in macrophages, along with a decrease in DNM2 (DYNAMIN 2) levels (Figure 3D,E). iNOS is commonly associated with classically activated M1 macrophages, which are typically linked to antitumor immune responses, whereas ARG1 is a marker of alternatively activated M2 macrophages, which are often associated with immunosuppressive functions.^[^ ^29^ ^]^ The simultaneous upregulation of both iNOS and ARG1 suggests that these macrophages exhibit features of a mixed M1/M2 phenotype, a state frequently observed in TAMs within the tumor microenvironment.^[^ ^30^, ^31^ ^]^ Furthermore, the observed downregulation of DYNAMIN 2, a GTPase required for effective phagocytosis, may indicate impaired macrophage function in response to eIF4E‐EVs.^[^ ^32^, ^33^ ^]^ Similar findings were validated in THP1‐derived macrophages (Figure S3A,B, Supporting Information).

Seahorse assays, probing glycolytic and mitochondrial functions, demonstrated the metabolic shifts triggered by eIF4E‐EVs in macrophages. Intriguingly, peritoneal macrophages treated with eIF4E‐EVs showcased heightened extracellular acidification rates (ECAR) relative to the control EV group (Figure 3F,G). Additionally, analysis of the oxygen consumption rate (OCR) unveiled a decline in mitochondrial respiration capacity among macrophages treated with eIF4E‐EVs (Figure 3H,I). These findings were consistently observed in THP1‐derived macrophages exposed to both control and eIF4E‐EVs (Figure S3C–F, Supporting Information). In contrast, treatment with eIF4A1‐EVs had no significant effect on ECAR and OCR when compared to control EVs (Figure S3G,H, Supporting Information). Additionally, a glucose consumption assay was conducted, where glucose‐deprived macrophages were cocultured with 2‐NBDG, a glucose analog. Peritoneal and THP1‐derived macrophages stimulated by eIF4E‐EVs showed a significant increase in glucose uptake compared to those treated with control EVs (Figure 3J and Figure S3I, Supporting Information). In addition to glycolysis assays, cholesterol was quantified in the EV‐treated macrophages, which revealed a significant increase in cholesterol levels following treatment with eIF4E‐EVs (Figure 3K and Figure S3J, Supporting Information). Conversely, macrophages treated with eIF4A1‐EVs exhibited no increase in glucose uptake and cholesterol levels compared to those treated with control EVs (Figure S3K,L, Supporting Information). This finding further corroborates the SILAC analysis, indicating no significant changes in the expression of genes associated with the glucose and cholesterol pathways upon eIF4A1‐EVs treatment. Consequently, we focused on determining the translational and metabolic alterations specifically induced by eIF4E‐EVs in macrophages for subsequent studies.

To elucidate the paracrine signaling interactions between cancer cells and TAM, ID8 *Trp53^−/−^;Brca2^−/–^* were cocultured with peritoneal macrophages treated with eIF4E‐EVs under various conditions for 48 h. Tumor cells cocultured with eIF4E‐EV treated macrophages exhibited significantly elevated cholesterol levels when compared to both the no EV and control EV groups (Figure 3L,M). Similarly, when OVCAR8 cells were cocultured with THP1‐derived macrophages treated with EVs, an increase in cholesterol levels in OVCAR8 cells within the eIF4E‐EVs group was observed (Figure S3M, Supporting Information). These collective findings underscore the pivotal role of tumor derived EVs packed with eIF4E in reprogramming macrophage metabolism.

### 2.4 eIF4E‐Enriched EVs Drive Metabolic and Translational Rewiring in Macrophages to Activate Cholesterol Synthesis

**Figure 4:**
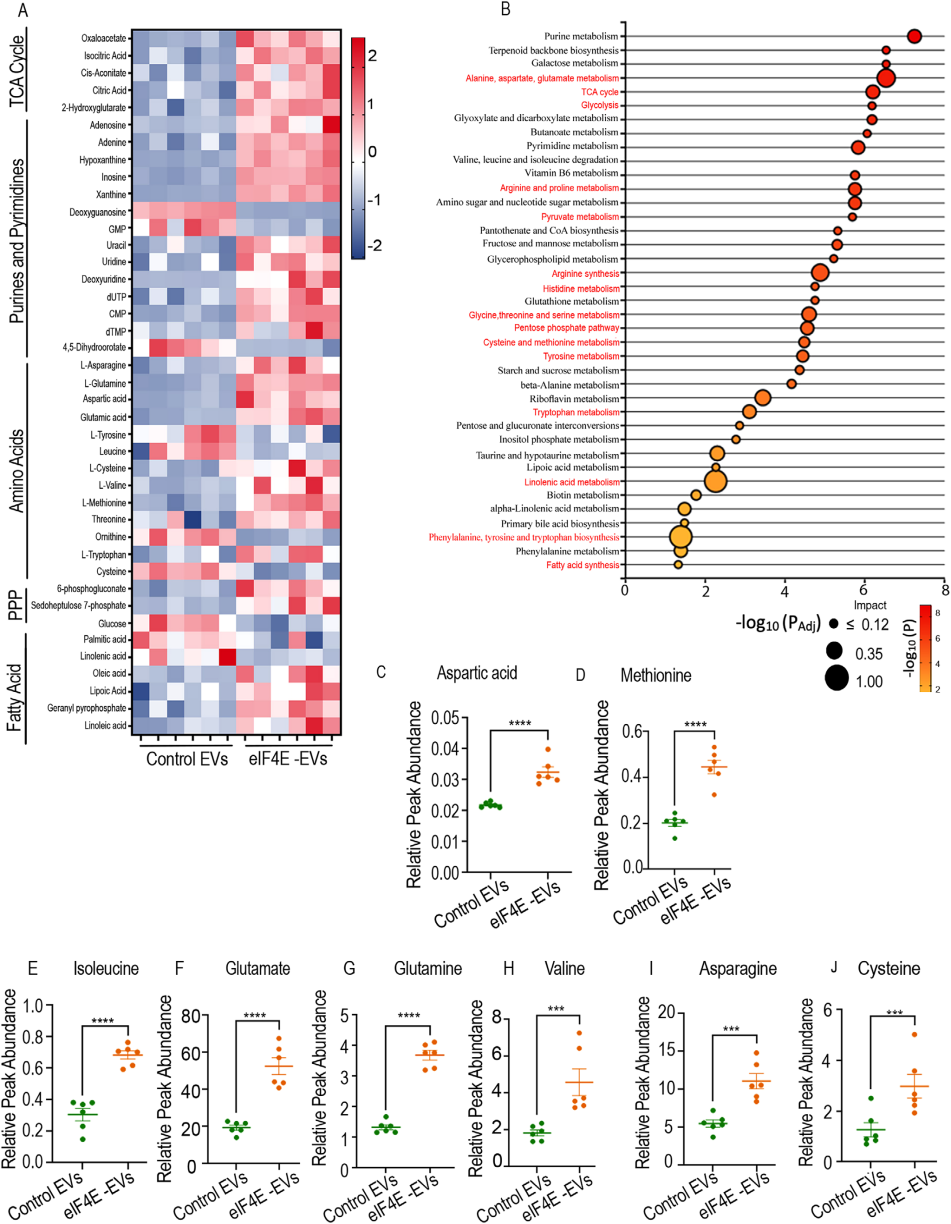
Effect of tumor derived EVs on global metabolomics. A) Heat‐map of untargeted metabolic analysis showing z‐scores of differentially upregulated and downregulated metabolites (*n* = 6). B) Pathway enrichment analysis based on human metabolic pathways in the kyoto encyclopedia of genes and genomes (KEGG) database (*n* = 6). C–J) Relative metabolite levels (peak intensity normalized by internal standard and cell number) between control and eIF4E‐EVs treated macrophages (*n* = 6). The data are shown as mean ± SEM. ***p < 0.001 ****p < 0.0001 compared to control by Student's *t*‐test and one‐way ANOVA.

The expression of enzymes associated with glucose, glutamine, and cholesterol metabolism was increased by treatment of macrophages with eIF4E‐EVs (Figure 3E). As protein synthesis directly depends upon the amino acid generation via the tricarboxylic acid (TCA) cycle, we wanted to check the incorporation of amino acids and their metabolites, as well as the consumption of amino acids toward protein synthesis after EVs treatment. Untargeted metabolic analysis was conducted on macrophages exposed to tumor derived EVs. Interestingly, our global metabolic assessments aligned with the upregulated protein synthesis in macrophages treated with eIF4E‐EVs. Specifically, elevated levels of essential amino acids (valine, methionine, threonine, tryptophan) and nonessential amino acids (asparagine, glutamine, aspartate, glutamate), which are important for protein synthesis during translation, were observed (**Figures** **4**A–J). The upregulation of protein translation is known to be coupled with nucleotide biosynthesis, facilitated by eIF4E mediated regulation of phosphoribosyl pyrophosphate synthetase (PRPS2).^[^ ^34^ ^]^ PRSP2 is a rate limiting enzyme of the pentose phosphate pathway, exerting control over nucleotide biosynthesis. Interestingly, our untargeted analysis unveiled an augmentation in pentose phosphate pathway intermediates, such as sedoheptulose 7‐phosphate and 6‐phosphogluconate. Additionally, an overall elevation in purines, pyrimidines, and intermediates of the TCA cycle in macrophages treated with eIF4E‐EVs was observed (Figure 4A,B and Figure S4A–M, Supporting Information). Intriguing changes in the lipid metabolism of macrophages treated with eIF4E‐EVs were also noted. Specifically, an increase in immunosuppressive polyunsaturated fatty acids (oleic and linoleic acid) alongside a decline in saturated fatty acid (palmitic acid) levels was observed. Consistent with our SILAC data analyses, elevated levels of geranyl pyrophosphate, a precursor in the cholesterol synthesis pathway, in eIF4E‐EV treated macrophages compared to control macrophages were also observed (Figure 4A,B). Overall, these systemic metabolic changes, combined with protein expression analysis, hinted at an increase in amino acid, nucleotide, and cholesterol biosynthesis in EVs treated macrophages.

**Figure 5:**
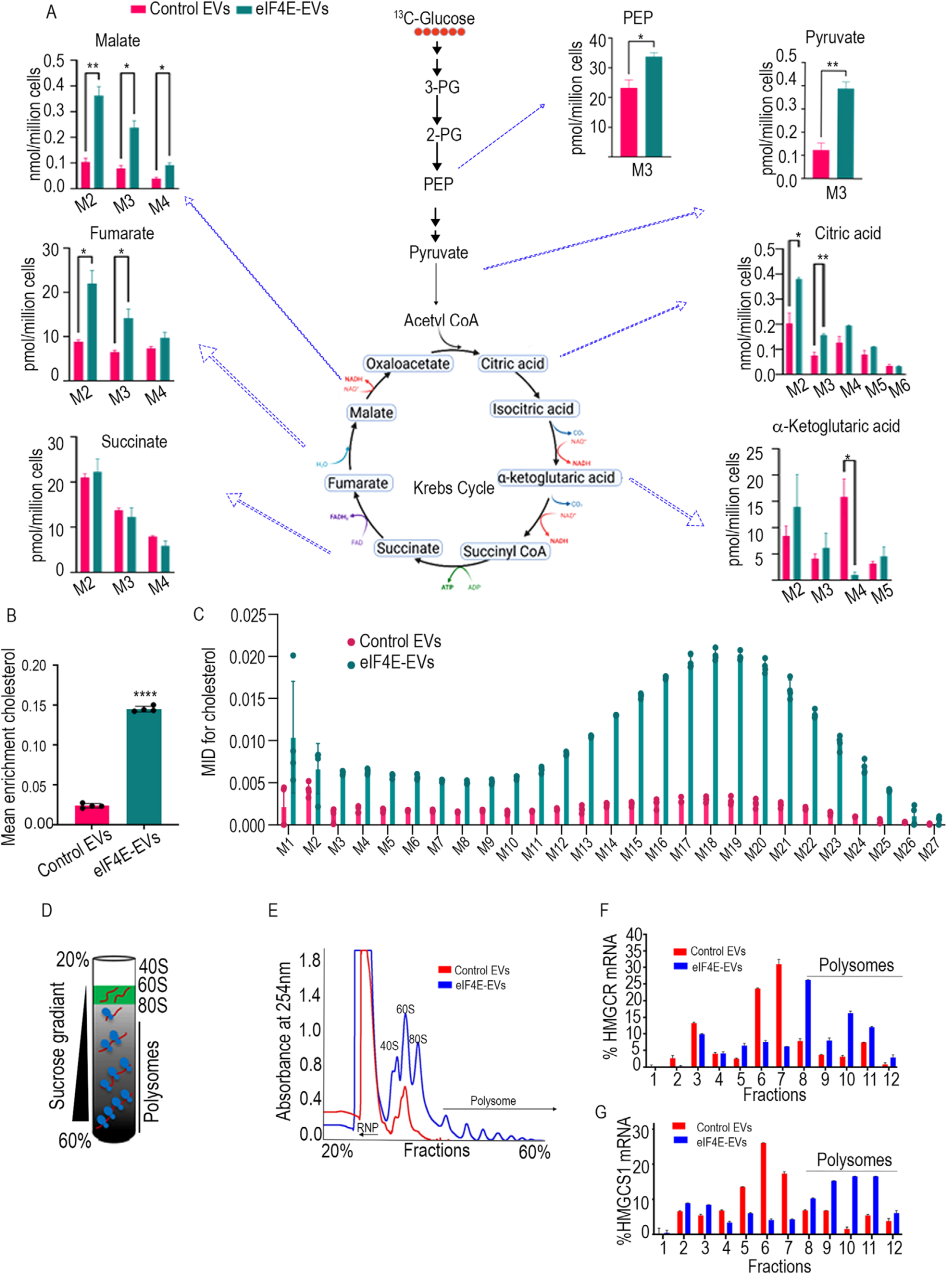
Tumor derived EVs with eIF4E encapsulation enhance de novo cholesterol synthesis in macrophages. A) Fractional contribution of glucose in central carbon metabolites in control and eIF4E‐EVs treated macrophages (*n* = 3). B) Mean enrichment showing the contribution of uniformly labeled ^13^C glucose in cholesterol (*n* = 4). C) Mass isotopologue distribution (MID) of cholesterol (*n* = 4). D) Schema representing polysome profiling. E) Plot of the absorbance profile of fractions obtained through sucrose gradients to isolate polysomes. F,G) qPCR shows the enrichment of HMGCR and HMGCS1 mRNA in the isolated fractions bound with free RNPs, monosomes, and polysomes. Data were normalized with the control group. The data are shown as mean ± SEM. *p < 0.01, **p < 0.05, compared to control by Student's *t*‐test and one‐way ANOVA.

Further, to unravel the contribution of glucose or glutamine metabolism toward lipogenesis, semitargeted stable isotope tracing analysis of EVs treated macrophages was done using gas chromatography mass spectrometry. ^13^C labeled glucose tracing revealed an increased amount of glycolytic and TCA cycle intermediates synthesized from glucose in EVs treated macrophages. More specifically, we noted elevated levels of M+3 phosphoenolpyruvic acid and pyruvate. A similar trend was seen in the TCA metabolites such as malate, fumarate, and citrate (**Figure** **5**A). Furthermore, several other metabolites, including alanine, aspartate, 2‐hydroxyglutarate, cytosine, and proline were also synthesized in greater amounts from labeled glucose (Figure S5A–E, Supporting Information). Therefore, it can be inferred that macrophages treated with eIF4E‐EVs are actively utilizing the glycolytic pathway for biosynthesis and adenosine triphosphate (ATP) generation as compared to control EVs. Conversely, glutamine tracing experiments revealed no significant difference in glutamine utilization between the two groups (Figure S5F, Supporting Information). To determine the source of the elevated cholesterol levels in treated macrophages (Figure 3K), ^13^C labeled glucose tracing was utilized for 96 h. Remarkably, an increase in de novo cholesterol synthesis was observed, as evidenced by the heightened incorporation of labeled glucose into cholesterol in macrophages treated with eIF4E‐EVs (Figure 5B,C).

Based on our previous findings, which demonstrated that eIF4E‐ EVs enhance global protein synthesis (Figure 2O), our objective was to investigate their direct impact on translation efficiency. To achieve this, macrophages were treated with EVs isolated from parental and OVACR8 cells overexpressing eIF4E and prepared cytoplasmic fractions. These fractions were further fractionated using 20–60% linear sucrose gradient columns (Figure 5D). The absorbance at 254 nm was recorded to profile polysome content, revealing four distinct peaks representing 40S and 60S ribosomal subunits, 80S monosomes, and polysomes from left to right. Notably, our assay detected the presence of elevated polysomal mRNA peaks, indicative of active translation, following the treatment of macrophages with eIF4E‐enriched EVs compared to control EVs (Figure 5E). Proteins from gradient fractions were isolated and analyzed via sodium dodecyl sulfate ‐polyacrylamide gel electrophoresis (SDS‐PAGE); compared to the control EVs, a significant increase in the expression level of ribosomal protein RPS6 (S6) in response to eIF4E overexpression mediated by EVs was observed (Figure S5G,H, Supporting Information). Subsequently, in another experiment, the abundance of HMGCR and HMGCS1 mRNA in polysome fractions was determined by real‐time polymerase chain reaction (PCR) on mRNA purified from the ribosomal fractions. Consistent with our previous findings showing the translational enhancement induced by eIF4E‐EVs, we observed increased levels of HMGCR and HMGCS1 mRNAs in polysome fractions. (Figure 5F,G). This indicates that these transcripts are actively translated by eIF4E enriched EVs and contribute to EV‐mediated metabolic reprogramming in recipient cells.

### 2.5 Tumor Derived EVs with eIF4E Promote Tumor Growth and Drive an Immunosuppressive Phenotype in Macrophages

**Figure 6:**
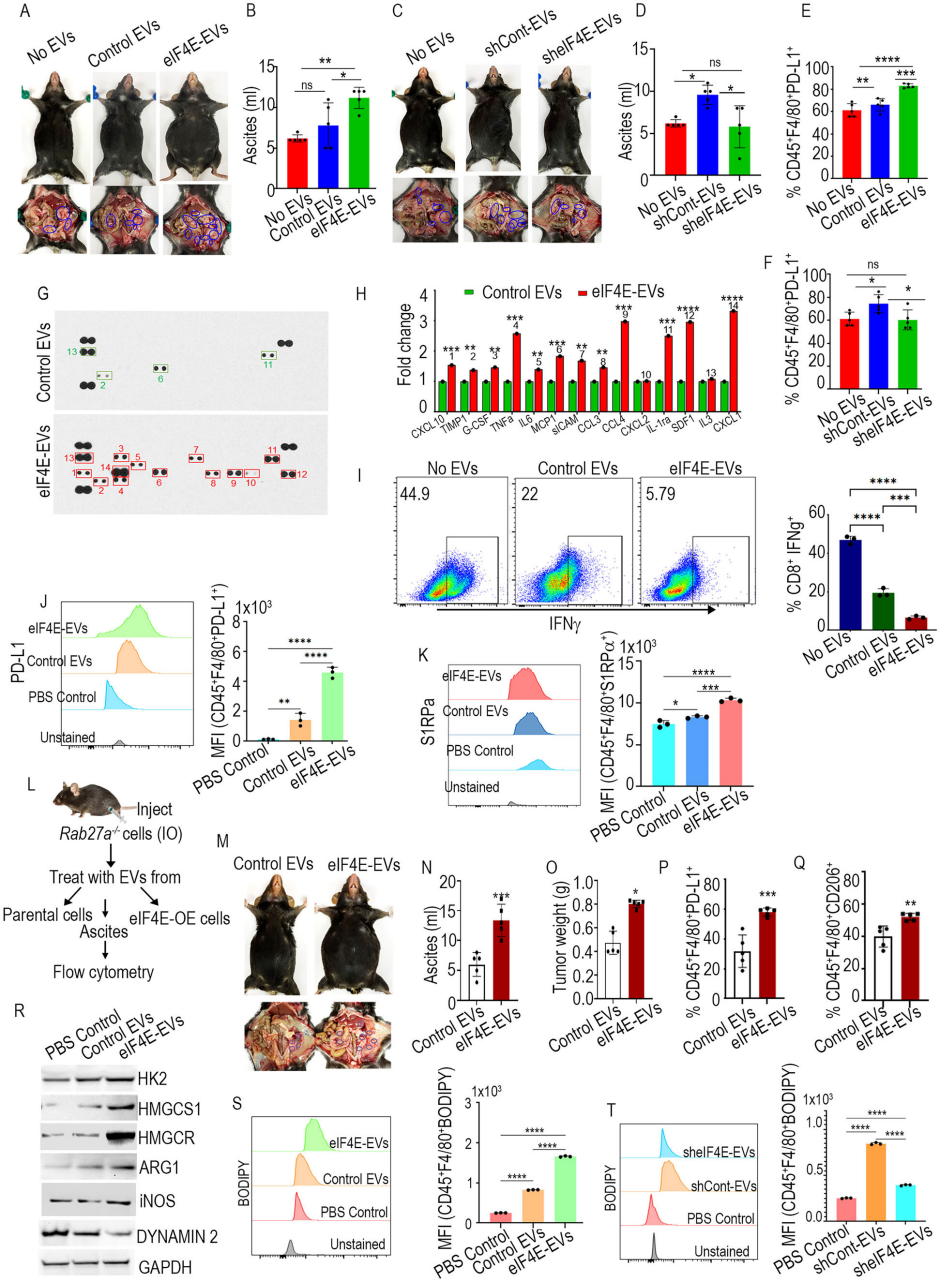
Tumor derived EVs enhance tumor growth and elevate PD‐L1 expression in macrophages. A) Peritoneal cavity of mice showing ascites accumulation and tumor locations (blue circles) (*n* = 5). B) Bar graph representing average ascites volume. C) Peritoneal cavity of the mice showing ascites accumulation and tumor locations (blue circles) (*n* = 5). D) Bar graph representing average ascites volume. E,F) Bar graphs representing PD‐L1 expression on macrophages in ascites samples. G) Representative cytokine array panels of supernatants taken from peritoneal macrophages stimulated with control EVs and eIF4E‐EVs (*n* = 3). H) Representative plots and the summarized mean pixel density of each protein. I) IFN‐γ production in activated CD8^+^ T cells (*n* = 3). J) Expression of PD‐L1 on peritoneal macrophages stimulated with EVs (*n* = 3). K) Expression of SIRPα on peritoneal macrophages (*n* = 3). L) Schema for experimental design (M–Q). M–O) Peritoneal cavity of the mice showing ascites accumulation and tumor locations (blue circles). (*n* = 5). P) PD‐L1 and Q) CD206 expression on macrophages in ascites (*n* = 5). R) Western blots showing expression of HK2, HMGCS1, ARG‐1, iNOS, and DYNAMIN 2 in macrophages. S,T) Bodipy staining on macrophages isolated from ascites (*n* = 3). The data are shown as mean ± SEM. *p < 0.01, **p < 0.05, ***p < 0.001, ****p < 0.0001 compared to control by Student's *t*‐test and one‐way ANOVA.

EVs exhibit the ability to disseminate to various tissues beyond their originating cells. Considering this, our initial investigation focused on the impact of ovarian cancer derived EVs on tumor progression. To accomplish this, C57BL/6 mice were injected with ID8 *Trp53^−/−^;Brca2^−/−^* cells into the ovary. After 7 d, mice were treated thrice a week intravenously with phosphate buffer saline (PBS) (no EVs control) or with 20 µg EVs isolated from: i) ID8 *Trp53^−/−^;Brca2^−/−^* cells, ii) ID8 *Trp53^−/−^;Brca2^−/−^* cells overexpressing eIF4E, or iii) eIF4E knock down ID8 *Trp53^−/−^;Brca2^−/−^* with appropriate controls. The EV concentrations were selected based on previously published literature.^[^ ^35^, ^36^ ^]^ Mice treated with eIF4E‐enriched EVs displayed augmented tumor nodules and increased ascites compared to those injected with control EVs and no EVs (**Figures** **6**A,B and S6A,B, Supporting Information). Conversely, mice administered with EVs derived from eIF4E KD ID8 *Trp53^−/−^;Brca2^−/−^* cells exhibited decelerated tumor growth when compared to those receiving EVs from ID8 *Trp53^−/−^;Brca2^−/−^* cells (Figure 6C,D and Figure S6C,D, Supporting Information).

Moreover, flow cytometry analysis of the ascites demonstrated an enhanced expression of programmed death ligand 1(PD‐L1) and CD206 on F4/80^+^ TAM in mice treated with eIF4E‐EVs (Figure 6E and Figure S6E, Supporting Information). The group treated with eIF4E knockdown EVs exhibited a significant decrease in the expression of PD‐L1 and CD206 on F4/80^+^ cells (Figure 6F and Figure S6F, Supporting Information). These findings show that exogenously administered eIF4E‐packed EVs selectively induce the upregulation of CD206 and PD‐L1 expression in macrophages, thereby fostering the emergence of immunosuppressive macrophages within the tumor.^[^ ^37^, ^38^ ^]^ To investigate the impact of tumor derived eIF4E‐EVs on macrophage functionality, a cytokine array was conducted using the macrophage supernatant. Macrophages stimulated by eIF4E‐EVs displayed significant increases in the secretion of tumor necrosis factor ‐α (TNF‐α), IL6, MCP1, CXCL1, IL‐1ra, CCL4, and SDF1 compared to those treated with control EVs (Figure 6G,H). To directly assess the impact of eIF4E‐EVs‐stimulated macrophages on effector T cell function, peritoneal macrophages were pretreated with either control or eIF4E‐EVs before coculturing them with activated murine T cells. CD8^+^ T cells exhibited a significant reduction in interferon‐γ (IFN‐γ) production (Figure 6I), indicating eIF4E‐EVs stimulation polarizes macrophages toward an immunosuppressive phenotype.

Consistent with the in vivo phenotype, peritoneal macrophages isolated from naïve mice and treated in vitro with eIF4E‐EVs exhibited increased surface expression of PD‐L1 compared to those treated with control EVs or untreated (Figure 6J).

However, it remains unclear whether EV stimulation induces endogenous PD‐L1 expression in macrophages or if PD‐L1 is passively transferred from the EVs. Western blot analysis confirmed that EVs derived from ID8 *Trp53^−/−^;Brca2^−/−^* cells express PD‐L1, consistent with previous reports (Figure S6G, Supporting Information).^[^ ^39^, ^40^ ^]^ Furthermore, macrophages stimulated with eIF4E‐EVs exhibited elevated PD‐L1 expression compared to those treated with control EVs, sheIF4E‐EVs, or untreated macrophages. Notably, the level of PD‐L1 present in ID8 *Trp53^−/−^;Brca2^−/−^* EVs was insufficient to account for the PD‐L1 expression observed in stimulated macrophages (Figure S6H, Supporting Information), indicating that the upregulation of PD‐L1 is primarily due to de novo synthesis. Apart from the widely recognized PD‐1/PD‐L1 axis, another pivotal interaction emerging in the field of antitumor immunity is the interplay between CD47 and SIRPα.^[^ ^41^, ^42^, ^43^ ^]^ The expression level of SIRPα on eIF4E‐EVs stimulated macrophages increased compared to those treated with control EVs or vehicle (Figure 6K). These data show that tumor‐derived EV stimulation skews macrophages toward an immunosuppressive phenotype, involving both direct PD‐L1‐mediated and indirect cytokine‐mediated mechanisms. Subsequently, our objective was to demonstrate that tumor derived EVs are responsible for upregulating PD‐L1 expression in macrophages, thereby facilitating the priming of ovarian cancer metastasis. To address this question, CRISPR/Cas9 technology was used to silence *Rab27a*, a critical regulatory protein essential for EV secretion.^[^ ^44^, ^45^ ^]^ In brief, *Rab27a^−/–^* ID8 cells were injected into the ovary of female C57BL/6 mice, resulting in tumors incapable of releasing EVs. Ten days following tumor cell injection, the mice were randomized into three groups and treated with EVs isolated from i) ID8 *Trp53^−/−^;Brca2^−/−^* cells, ii) ID8 *Trp53^−/−^;Brca2^−/–^* cells that overexpressed eIF4E, or iii) eIF4E knock down ID8 *Trp53^−/−^;Brca2^−/−^* cells. The mice were closely monitored for tumor progression. The administration of EVs obtained from cells that overexpressed eIF4E increased tumor weight, ascites volume, and body weight (Figure 6L–O and Figure S6I, Supporting Information). Additionally, there was no significant difference in the total number of macrophages in ascites samples from tumor bearing mice treated with either control or eIF4E‐EVs (Figure S6J,K, Supporting Information). However, these macrophages displayed notable phenotypic alterations, evidenced by increase in PD‐L1 and CD206 expression in eIF4E‐EVs group, despite comparable overall counts (Figure 6P,Q) In contrast, the number of macrophages in peritoneal wash samples from naïve mice was significantly lower than in tumor bearing mice, suggesting that tumor progression promotes both macrophage recruitment and phenotypic reprogramming (Figure S6L, Supporting Information).

In another independent experiment, mice injected with EVs derived from eIF4E knockdown cells exhibited reduced tumor burden as well as lower levels of CD206 and PD‐L1 expression on macrophages compared to those treated with EVs from ID8 *Trp53^−/−^;Brca2^−/−^* cells (Figure S6M–S, Supporting Information).

To investigate the impact of eIF4E‐EVs on macrophage metabolism in vivo, TAMs were isolated from ascites by fluorescence activated cell sorter (FACS), followed by western blot analysis. In line with our earlier in vitro experiments on peritoneal macrophages from naïve mice (Figure 3E), macrophages isolated from eIF4E‐EVs treated mice showed increased expression levels of ARG 1, HK2, GLS, HMGCS1, HMGCR, iNOS, and a reduction in DYNAMIN 2 levels (Figure 6R) compared to macrophages isolated from control EV treated mice. Furthermore, TAMs isolated from mice treated with eIF4E‐EVs had the highest lipid content compared to control EVs (Figure 6S). Conversely, TAMs isolated from mice treated with sheIF4E‐EVs displayed lower lipid content compared to control EVs (Figure 6T).

Apart from that, there was a significant reduction in the overall tumor burden and PD‐L1 expression in the *Rab27a^−/−^* group compared to the control (Figure S6T‐SX, Supporting Information). These findings collectively confirm that eIF4E‐EVs specifically impact macrophage metabolism to promote tumor growth.

### 2.6 Targeting HMGCR in Macrophages Enhances Antitumor Immune Response

**Figure 7:**
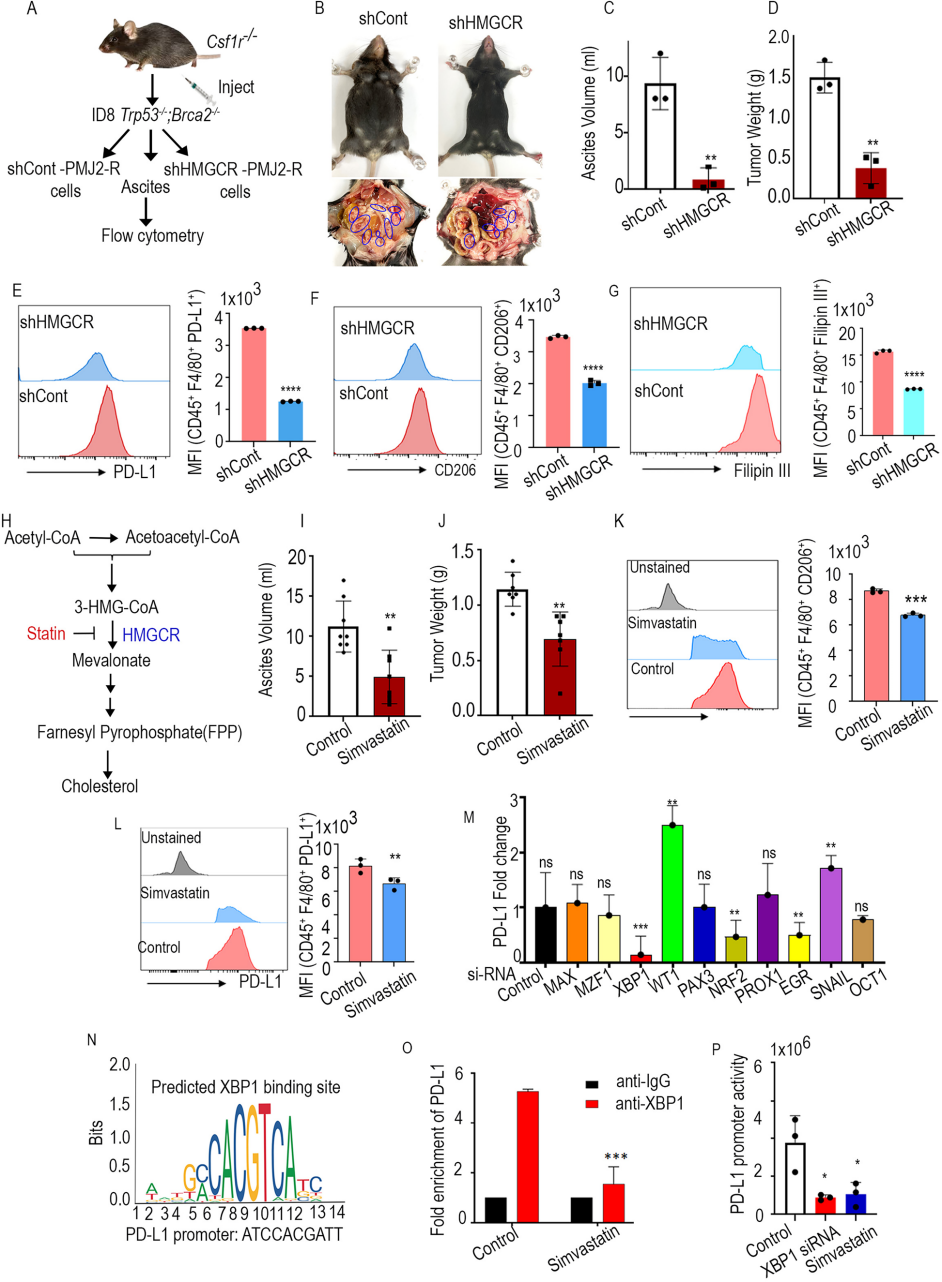
Inhibition of cholesterol synthesis enhances antitumor immune responses. A) Schematic workflow. B) The peritoneal cavity of mice showing ascites accumulation and tumor locations (*n* = 3). C) Bar graphs representing ascites volume and D) tumor weight. E) PD‐L1, F) CD206, and G) Filipin III expression on macrophages in ascites samples (*n* = 3). H) Schema showing the cholesterol biosynthesis pathway. I) Bar graph representing ascites volume and J) tumor weight in tumor bearing mice treated with control and simvastatin (*n* = 7). K,L) Flow cytometric quantification of the CD206^+^ and PD‐L1^+^ macrophages in ascites. M) Validation of transcription factor array using qPCR (*n* = 3). N) Predicted XBP1s binding sites on PD‐L1 promoters. O) ChIP analysis of XBP1s binding to PD‐L1 promoters in macrophages treated with simvastatin (*n* = 3). P) Dual luciferase analysis of the effect of XBP1 KD and simvastatin treatment on PD‐L1 promoter in macrophages (*n* = 3). Error bars indicate mean ± SEM. *p < 0.05, **p < 0.01 ****p < 0.0001. ns, nonsignificant.

To determine the impact of reducing cholesterol levels in macrophages on their antitumor activity, either control or Hmgcr‐KD (shHMGCR)‐PMJ2‐R cells were injected into *Csf1r^−/–^* C57BL/6 mice bearing ID8 *Trp53^−/−^;Brca2^−/–^* tumors (**Figure** **7**A,B). Mice treated with shHMGCR‐PMJ2‐R cells displayed reduced ascites volume, tumor weight, and body weight when compared to the control mice (Figure 7C,D and Figure S7A, Supporting Information). Tumor infiltrating shHMGCR‐PMJ2‐R cells in tumor‐bearing mice exhibited decreased expression of PD‐L1 (Figure 7E) and CD206 (Figure 7F) as well as lower cholesterol content when compared to control PMJ2‐R tumor bearing mice (Figure 7G). Given that the TAMs and the TME often exhibit elevated cholesterol levels in comparison to normal cells or tissues, we sought to determine whether reducing cholesterol levels in a tumor or its microenvironment would impact the expression of immune checkpoint markers on macrophages in an in vivo setting. To assess the effects of pharmacological inhibition of cholesterol on tumor growth and macrophage activation, *ID8 Trp53^−/−^;Brca2^−/–^* cells were orthotopically injected into syngeneic C57BL/6 mice and treated with HMGCR inhibitor simvastatin (Figure 7H and Figure S7B, Supporting Information). Simvastatin treatment reduced the ascites and tumor weight when compared to the control mice (Figures 7I,J and Figure S7C, Supporting Information). Moreover, simvastatin treatment led to decreased expression of CD206 and PD‐L1 on macrophages isolated from ascites, in contrast to control mice (Figure 7K,L). Furthermore, CD8^+^ T cells from simvastatin‐treated mice displayed significantly higher Ki67 and increased expression of granzyme B, the executor of tumor cell killing (Figure S7D–F, Supporting Information). IHC analysis revealed a marked reduction in Ki67 levels in tumor tissue isolated from mice treated with simvastatin, in comparison to control mice (Figure S7G, Supporting Information). These findings highlight the role of cholesterol in immunosuppressive reprogramming and tumor progression. To confirm these results at the cellular level, the impact of simvastatin treatment on peritoneal macrophages was assessed, which exhibited a reduction in PD‐L1 expression (Figure S7H, Supporting Information). To further gain insights into the underlying mechanism of statin treatment and its impact on PD‐L1 downregulation, a transcription factor (TF) array was employed. Macrophages were treated with simvastatin, and nuclear extracts were collected for the TF profiling assay. Simvastatin treatment led to a 2.5‐fold reduction in the expression of several transcription factors, including early growth response (EGR), XBP1, MZF, NRF‐2, OCT‐1, Pax‐3, MEF1, and Prox1 (Figure S7I, Supporting Information).

To further confirm the involvement of these transcription factors, we used target‐specific siRNAs to knock them down in macrophages, followed by quantitative polymerase chain reaction (qPCR) analysis of PD‐L1 expression. We found that loss of XBP1 led to a reduction in PD‐L1 expression (Figure 7M). Subsequently, the mechanism by which XBP1 regulates the PD‐L1 expression on macrophages was explored. Given that XBP1 is a transcription factor,^[^ ^46^ ^]^ PD‐L1 gene promoters were analyzed and potential binding sites for XBP1 were identified (Figure 7N). We then assessed whether simvastatin treatment could attenuate XBP1's binding to the PD‐L1 promoter by chromatin immunoprecipitation (ChIP) assay. Simvastatin‐treated macrophages had reduced binding of XBP1 on the PD‐L1 promoter compared with untreated macrophages (Figure 7O). Furthermore, luciferase reporter assay showed that both XBP1 silencing and simvastatin treatment led to a decrease in PD‐L1 promoter activity compared to the control group, indicating that XBP1 activates PD‐L1 transcription (Figure 7P). Collectively, our data establish that cholesterol contributes to the immunosuppressive phenotype of macrophages.

### 2.7 HMGCR Expression in TAMs Is Associated with Poor Prognosis of Ovarian Cancer Patients

**Figure 8:**
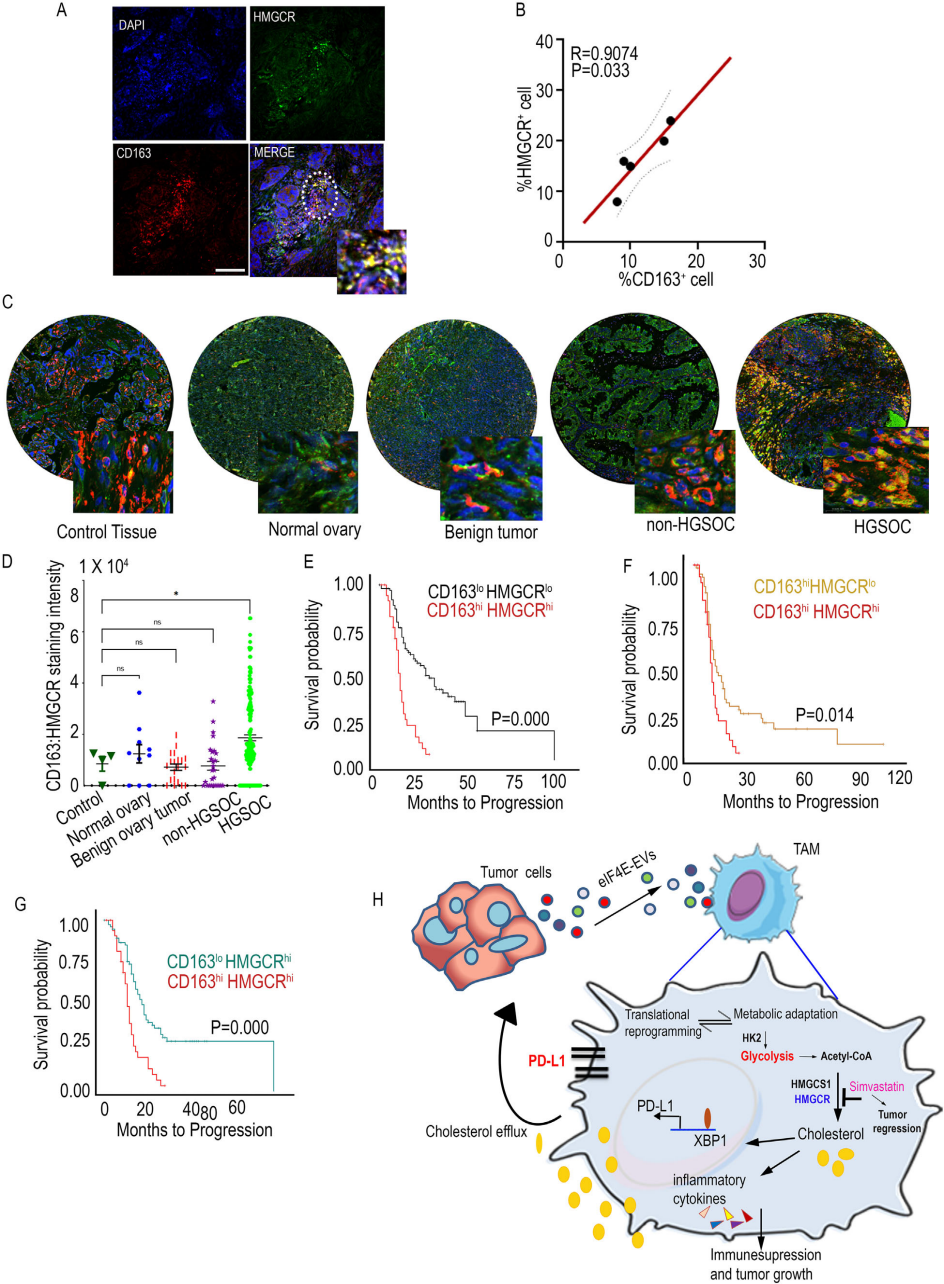
Increased HMGCR expression in TAM predicts poor prognosis. A) Representative IF staining of HMGCR (Green) and CD163 (red) in human Ovarian cancer tissue. Bar 50 µm (*n* = 5). B) Correlation analysis of CD163^+^/HMGCR^+^ cell proportions in(A). C,D) Co‐immunofluorescence staining of HMGCR (Green) and CD163 (red) in human ovarian cancer tissue microarray. E) Kaplan‐Meier survival curves generated from the ovarian cancer patient dataset based upon the expression profile of HMGCR and CD163. High HMGCR and high CD163 expression (*n* = 35) versus low HMGCR and low CD163 expression (*n* = 97). F) High HMGCR and high CD163 expression (*n* = 35) versus low HMGCR and high CD163 expression (*n* = 64). G) High HMGCR and high CD163 expression (*n* = 35) versus high HMGCR and low CD163 expression (*n* = 78). H) Diagram illustrating how eIF4E loaded EVs reprogram macrophage metabolism and induce immunosuppression. Error bar represents ± SEM, *p < 0.05, ***p < 0.001, (Student's *t*‐test).

Our in vivo experiments demonstrated that elevated HMGCR expression on TAMs played a protumorigenic role. To assess whether a similar phenomenon occurs in ovarian cancer patients, tumor tissue was obtained from high‐grade ovarian cancer patients. Immunofluorescence (IF) staining for HMGCR and CD163, a marker for TAMs, revealed that HMGCR is expressed on CD163^+^ cells (**Figure** **8**A). Correlation analysis showed that the expression of HMGCR in macrophages was proportional to the number of CD163^+^ cells (Figure 8B). We thus speculated that high HMGCR expression was related to intertumoral TAM aggregation in ovarian cancer. To test this hypothesis, the potential codistribution between HMGCR and TAMs was investigated in serial sections of ovarian cancer tissue microarrays of 252 cores by IF staining, and increased coexpression of HMGCR and CD163 in malignant high‐grade samples was found (Figure 8C,D and Figure S8A,B, Supporting Information). To further explore the clinical significance of HMGCR upregulation and TAM recruitment in ovarian cancer, survival analyses were conducted for associated genes. Clinical data were obtained from a publicly available ovarian cancer dataset by Tothill et al.^[^ ^47^ ^]^ Our analysis revealed that the expression of CD163 and CD206 alone did not significantly impact overall survival (Figure S8C,D, Supporting Information). However, patients exhibiting high expression levels of both CD163 and HMGCR had a poorer survival compared to those with low expression levels (Figure 8E). Additionally, in patients with high CD163 expression, better survival outcomes were observed in those with low HMGCR expression (Figure 8F). Similarly, in patients with high HMGCR expression, better survival outcomes were observed in those with low CD163 expression (Figure 8G). Survival analyses of CD206 and HMGCR expression also yielded consistent results (Figure S8E–G, Supporting Information). Collectively, these findings suggest that HMGCR‐positive TAMs are linked to a worse prognosis in ovarian cancer patients. This supports the notion that HMGCR signaling is elevated in TAMs, potentially contributing to the initiation and progression of ovarian cancer.

## 3 Discussion

Extracellular vesicles produced by cancer cells serve as a distinctive mode of intercellular communication, exerting influence on cell growth and survival, and helping shape the tumor microenvironment. These EVs carry various cargoes, including mRNA, miRNA, proteins, and transcription factors, which can modulate gene expression and cellular functions in both normal and cancer cells. Studies have established a direct correlation between the presence of EVs and gene regulation.^[^ ^48^, ^49^ ^]^ While the contents of EVs are characterized as both diagnostic and prognostic biomarkers, the impact of EVs on tumor resident immune and stromal cells is not well characterized. Importantly, our study provides evidence on how EVs dysregulate the function of T cells and create an immunosuppressive environment due to translational adaptations in tumor resident macrophages.

To further explore the impact of EVs content, we used an unbiased mass spectrometric approach to identify the proteins expressed in EVs. Strikingly, we found that the EVs derived from tumor cells exhibit elevated levels of translation initiation factor eIF4E. Mechanistically, we demonstrated that eIF4E interacts with the UEV domain of TSG101, facilitating its encapsulation into EVs.

eIF4E protein is a critical part of the eukaryotic translation initiation factor 4F complex, which recognizes the 7‐methylguanosine cap structure at the 5′ end of messenger RNAs and recruits ribosomes to the 5′‐cap structure. eIF4E, a cap‐binding protein, collaborates with proteins like eIF4A and eIF4G to bind mRNA, facilitating ribosome recruitment and translation initiation.^[^ ^50^ ^]^ In brief, the association of eIF4E with the 4F complex is considered the rate‐limiting step in translation initiation.^[^ ^51^, ^52^ ^]^ Notably, increased expression of eIF4E has been linked to various oncogenic activities in ovarian cancer cells, including proliferation, migration, invasion, and chemosensitivity.^[^ ^52^, ^53^ ^]^ Supporting the notion that eIF4E is associated with oncogenic functions, our data demonstrate how eIF4E influences the translation process in macrophages through the uptake of EVs loaded with eIF4E.

Metabolic interplay among immune cells, stromal cells, and cancer cells significantly impacts tumor evasion from immune surveillance, fostering cancer progression and metastasis.^[^ ^54^, ^55^ ^]^ Macrophages within the tumor microenvironment exhibit diverse metabolic programs, challenging the conventional M1–M2 classification.^[^ ^56^ ^]^ TAMs, which are associated with all stages of neoplastic progression in patients, comprise a large proportion of the immune infiltrate in malignancies. Ovarian cancer cells are known to promote cholesterol efflux in TAM by inducing IL‐4 mediated arginase programming.^[^ ^57^ ^]^

We elucidated how eIF4E‐packaged EVs affect metabolic profiles in macrophages within the tumor microenvironment. Importantly, eIF4E uptake in macrophages induced the translation of ribosomal proteins and key enzymes HMGCR and HMGCS1 involved in cholesterol synthesis. Metabolic analysis also revealed that eIF4E uptake in macrophages enhanced nucleotide biosynthesis, increased pentose phosphate pathway activity, increased the levels of enzymes involved in glucose, glutamine, and cholesterol metabolism, and elevated levels of amino acids essential for protein synthesis.

We further observed that the metabolic adaptations promote the immunosuppressive characteristics of macrophages, such as high expression of ARG 1, iNOS, and PD‐L1. We also observed the expression of both proinflammatory cytokines and anti‐inflammatory cytokines, indicating that eIF4E‐EV‐educated macrophages represent a mixed M1/M2 phenotype.^[^ ^29^, ^30^, ^31^ ^]^

Macrophages treated with eIF4E‐EVs displayed reduced expression of DYNAMIN 2 and elevated levels of SIPRα.^[^ ^41^, ^42^ ^]^ The latter has been implicated in the suppression of phagocytic activity in macrophages. Together, these findings suggest that eIF4E‐EVs induced a phenotype characterized by reduced phagocytic capacity, although functional validation will be required in future studies. Importantly, our study demonstrates that inhibiting EV secretion reduces PD‐L1 expression on TAMs in ovarian tumor bearing mice, highlighting the role of eIF4E in modulating PD‐L1 expression and influencing metastasis in ovarian cancer. While miRNA‐mediated regulation is a well‐established mechanism by which cancer cell derived EVs can modulate PD‐L1 expression in recipient immune cells,^[^ ^58^ ^]^ both in vitro and in vivo experiments consistently demonstrated that the observed increase in PD‐L1 expression in macrophages is primarily driven by EV‐mediated eIF4E signaling. This conclusion is supported by functional experiments showing that overexpression or knockdown of eIF4E in tumor cells leads to a corresponding increase or decrease in PD‐L1 expression in macrophages. While we cannot completely exclude the potential contribution of EV associated miRNAs, our data strongly support eIF4E‐dependent signaling as the predominant mechanism in this context.

Cholesterol‐lowering drugs, particularly statins, have broad clinical implications.^[^ ^59^, ^60^, ^61^ ^]^ Our study shows the potential therapeutic implications of these drugs, as they exhibit anticancer properties by reducing PD‐L1 expression in cancer cells and modulating cholesterol metabolism in macrophages via the XBP1 transcription factor. While our integrative approach combining transcriptomic profiling, motif enrichment analysis, and functional validation consistently identified XBP1 as a key regulator, we acknowledge that other TFs induced by simvastatin could also contribute to PD‐L1 regulation. Transcription factors such as STAT3, NF‐κB, and hypoxia inducible factor‐1α (HIF‐1α) have previously been implicated in this process, and their potential involvement in our model cannot be excluded.^[^ ^62^, ^63^, ^64^ ^]^

Further studies, employing combinatorial knockdown or rescue approaches, will help elucidate possible cooperative or hierarchical interactions among these TFs. Nonetheless, the current data strongly support XBP1 as a dominant and direct regulator of PD‐L1 expression in this context. Overall, our study uncovered an overlooked oncogenic mechanism involving eIF4E‐packaged EVs that upregulate cholesterol synthesis in macrophages, fostering immune suppression within the tumor microenvironment. To determine if HMGCR expression in TAM is associated with poor prognosis and tumor progression, we checked the expression of HMGCR and CD163 in ovarian cancer patients. We found a positive correlation between HMGCR and M2 TAM‐related molecules in ovarian cancer.

Our study has some limitations that suggest directions for future research. Initially, although our results showed that TAMs exhibit high HMGCR and PD‐L1 expression and enhance tumor progression, identifying specific TAM subpopulations driving ovarian cancer progression is still needed. Future work will use single‐cell and phenotypic analyses of ovarian cancer samples with varying stages to pinpoint these subpopulations. Additionally, a combination of simvastatin and anti‐PD1 antibody therapy is needed to assess the efficacy and mechanisms underlying this combinatorial strategy in ovarian cancer. Our findings also revealed elevated expression of eIF4E in plasma‐derived EVs from ovarian cancer patients. These eIF4E‐enriched EVs could hold potential as biomarkers for predicting treatment response. The predictive power of this EV‐packaged eIF4E can be further enhanced by integrating them with multimodal data, such as genomic, transcriptomic, and proteomic profiles. Future research should explore how pretreatment plasma EV eIF4E levels, combined with other molecular datasets, can refine treatment stratification and guide personalized therapeutic strategies in ovarian cancer.

In summary, our study provides evidence that eIF4E‐loaded EVs derived from tumor cells induce translational reprogramming and increase the expression of HMGCR in macrophages. These findings have implications for the development of new treatments targeting cholesterol metabolism in macrophages to enhance the efficacy of cancer immunotherapies.

## 4 Resource Availability

### 4.1 Lead Contact

Further information and requests for resources and reagents should be directed to and will be fulfilled by the lead contact, Sunila Pradeep (spradeep@mcw.edu).

### 4.2 Materials Availability

This study did not generate new unique reagents.

### 4.3 Data and Code Availability

This paper does not report the original code. Any additional information required to reanalyze the data reported in this paper is available from the lead contact upon request.

## 5 Experimental Model and Subject Details

### 5.1 Patient Samples

Peripheral blood was collected from ovarian cancer patients and healthy individuals. Before participation, all individuals provided written informed consent under an Institutional Review Board‐approved protocol at the Medical College of Wisconsin (Approval No‐PRO41599). All the clinical samples were obtained from adult females and were deidentified prior to analysis. No personally identifiable information was accessible to the investigators.

### 5.2 Cell Lines

HeyA8 human ovarian cancer cells were purchased from the Characterized Cell Line repository at MD Anderson Cancer Center (Houston, TX, USA). IOSE‐21, OVCAR4, A2780, OVCAR5, and OVCAR8 cells were purchased from the National Cancer Institute (NCI). NIH‐OVCAR3 cells were purchased from American type culture collection/Physics science oncology center network bioresource core facility (ATCC/PBCF) repository. THP1, PMJ2‐R and RAW264.7 cell lines were purchased from ATCC. OVSAHO and Kuramochi cells were received from Taru Muranen at Beth Israel Deaconess Medical Center (Boston, Massachusetts, USA). The BR‐Luc murine cell lines were received as a kind gift from Sandra Orsulic (University of California, Los Angeles, CA). The ID8 *Trp53^−/−^;Brca2^−/−^* murine ovarian cancer cell line was a kind gift from Dr. Iain A. McNeish (Wolfson Wohl Cancer Research Centre, Institute of Cancer Sciences, University of Glasgow, Glasgow, United Kingdom). HEK293‐T cells were procured from Thermo Fisher Scientific. LP3 and GM07213 cells were procured from Coriell Institute for Medical Research (NJ, USA). RF24 cells were received from Dr. Arjan W. Griffioen (VU University Medical Center, Amsterdam, Netherlands). OSE cells were cultured by scraping the surface epithelium of normal ovarian tissues obtained from two patients with benign gynecologic pathology. ID8 *Trp53^−/−^;Brca2^−/−^* cells were cultured in Dulbecco's modified Eagle's medium (DMEM) (Sigma‐Aldrich) with 4% fetal bovine serum (FBS) (Atlanta Biologicals, GA, USA), and 1x ITS. NIH OVCAR3 cells were cultured in Roswell park memorial institute‐1640 (RPMI‐1640) Medium with 10% FBS (Atlanta Biologicals, GA, USA) and N‐2‐hydroxyethylpiperazine‐N'‐2‐ethanesulfonic acid (HEPES) (ATCC). OSE cells were cultured in Mammary epithelial cell growth medium (MEGM) media (Lonza, Basel, Switzerland) supplemented with 15% FBS. Fibroblast cells were cultured in Eagle's minimum essential medium with Earle's salts and nonessential amino acids and 10% nonheat inactivated FBS. LP3 cells were cultured in medium 199 and Microcarrier cell development buffer (MCDB) (1:1) supplemented with 15% nonheat inactivated FBS, 10 ng mL^−1^ epidermal growth factor (EGF), and 0.4 µg mL^−1^ hydrocortisone. THP1 and Raw264.7 cells were cultured in RPMI‐1640 medium with 10% FBS and HEPES. RF24 cells were cultured in Eagle's minimum essential medium supplemented with 10% FBS, nonessential amino acids, and modified Eagle medium vitamins. All other cell lines were cultured in DMEM (Sigma‐Aldrich) supplemented with 10% FBS and 1% Pen‐strep (Thermo Fisher Scientific Inc., Waltham, MA, USA) at 37 °C in a humidified incubator with 5% CO_2_. The cell lines were authenticated by short tandem repeat profiling (IDEXX BioAnalytics) and tested for Mycoplasma using MycoSensor PCR Assay kit (Agilent, Santa Clara, CA)

### 5.3 Mice

C57BL/6 black female mice were purchased from Envigo, B6.Cg‐Csf1r<tm1.2Jwp>/J female mice were purchased from the Jackson Laboratory. All experiments complied with protocols approved by the Institutional Animal Care and Use Committee at the Medical College of Wisconsin (Ethical approval No. AUA6352).

## 6 Experimental Section

### EV Isolation, Characterization, and Quantification

The supernatant was collected from ovarian cancer cells, and noncancerous epithelial cells were grown to 80% confluency in serum‐free media for 48–72 h. EVs were purified and characterized from the supernatant as previously described.^[^ ^2^, ^65^ ^]^ To purify EVs using a sucrose gradient, the resulting supernatant underwent ultracentrifugation at 4 °C (Beckman Coulter, Optima XPN‐100). These EVs were resuspended in PBS and layered onto a sucrose gradient, followed by centrifugation. The isolated EV pellets were lysed, and total EV protein concentrations were determined by the bicinchoninic acid (BCA) protein Assay kit. To purify circulating EVs from blood, cell free plasma at 16 500 × *g* for 45 min was centrifuged first to pellet large membranous vesicles. EVs were then purified from the supernatant, using the Exosome Isolation Kit (Invitrogen, Cat# 4484450).

The EV size distribution and concentrations were determined by NTA, using the NanoSight LM10 instrument (Malvern Panalytical, Malvern, UK) with a 488 nm laser and NTA3.1 software. Purified EVs were fixed in 2% paraformaldehyde for transmission electron microscopy (EM) following the protocol of Bulreigh et al.^[^ ^66^ ^]^ and examined in a Hitachi H600 TEM (EM facility at the Medical College of Wisconsin, USA). To assess the uptake of EVs by different cells, EVs were labeled using DIL red fluorescent labeling dye (Invitrogen, Cat# V22885). The DIL‐labeled EVs were then incubated with cells at 37 °C. The uptake of labeled EVs was assessed using confocal laser scanning microscope (LSM 510; Zeiss, Oberkochen, Germany).

### Protein Extraction and Trypsin Digestion

EV pellets were resuspended in a solution containing 40% Invitrosol, 20% acetonitrile, and 100 mm ammonium bicarbonate followed by sonication. Total protein concentrations were measured, and cysteines were reduced and alkylated. Proteins were then digested with LysC and trypsin enzymes. Peptides were cleaned up using paramagnetic particles, and their concentrations were determined. Samples were diluted using acetonitrile and formic acid, with the addition of a peptide retention time calibration mixture.

### Mass Spectrometry Analysis

Peptides were dissolved and separated using a NanoElute ultrahigh‐performance liquid phase system. Each sample underwent analysis on a Thermo Scientific Orbitrap Fusion Lumos MS via 3 technical replicate injections using data‐dependent acquisition (DDA). MS data were analyzed using the Proteome Discoverer 2.4 platform, referencing the Swiss‐Prot_Human with isoforms version from 2019‐05‐01. Differentially expressed genes were assessed within biological pathways using the IPA software (Ingenuity Systems Inc). Fisher's exact test calculated the *p*‐value to determine a potentially significant association between differentially expressed proteins and specific functional categories. A *p*‐value < 0.05 was deemed statistically significant. Identified proteins were crossreferenced with exosome data available from the ExoCarta database (http://www.exocarta.org).

### Immunohistochemistry and Immunofluorescence

Slides were dewaxed in xylene and rehydrated through graded ethanol to distilled water. Antigen retrieval for the slide specimens was performed using IHC‐Tek epitope retrieval solution and steamer set (IHC World, LLC. Cat#1W‐1100). The slides were then immersed in 3% H_2_O_2_ for 10 min to quench endogenous peroxidase followed by blocking with 10% goat serum for 1 h. Slides were incubated with primary antibodies and then secondary antibodies. Slides were stained with 3,3'‐diaminobenzidine (DAB) and hematoxylin, dehydrated, mounted, and then cover‐slipped. For immunofluorescence, the sections were incubated for 1 h in the dark with fluorochrome‐conjugated secondary antibodies. Nuclei were stained using Antifade Fluorescence Mounting Medium with DAPI. Slides were digitally scanned using Panoramic 250 FLASH III scanner (3D HISTECH ltd. Version 2.0) and the slide Viewer software (3D HISTECH ltd. Version 2.0) was used to view and analyze images.

### Western Blotting

An equal amount of total protein was resolved on precast 4–12% SDS‐PAGE gels (Biorad, Hercules, CA, USA) and the protein was transferred onto polyvinylidene difluoride (PVDF) membranes as previously described.^[^ ^2^ ^]^ Membranes were incubated with the desired primary and secondary antibodies. Protein expression was detected with a chemiluminescence kit. Lists of the antibodies and reagents used in this study are given in the Supporting Information.

### siRNA Transfection

Predesigned siRNAs and universal control negative siRNAs were obtained from Sigma‐Aldrich. Transfections were performed using the Lipofectamine RNAiMAX transfection reagent (Thermo Fisher Scientific Inc., Waltham, MA, Cat# 13778150). At 48 h post‐transfection, cells were harvested for further analysis.

### Co‐Immunoprecipitation GFP Trap

OVCAR8 cells stably expressing either GFP fusion proteins or TSG101 variants were washed with PBS and lysed by adding 1X lysis buffer supplemented with protease inhibitor. Cleared lysates were incubated with GFP‐nanobody agarose (GFP‐Trap, Chromotek, RRID: AB_2631358). The beads were washed with ice‐cold Co‐IP washing buffer, and proteins were eluted in sample buffer by boiling at 95 °C for 10 min, followed by western blotting.

### Proximity Ligation Assay

The DuoLink In Situ Red Starter Kit Mouse/Rabbit (Sigma‐Aldrich, Cat# DUO92008,) was used to detect proximity between eIF4E/eIF4A1and TSG101 proteins according to the manufacturer's protocol. OVCAR8 cells were cultured in 8‐well chamber slides (ibidi USA, Madison, WI, USA) and cultured overnight. The slides were then washed with cold 1x PBS, fixed in 4% paraformaldehyde for 30 min, and blocked with Duolink Blocking Solution in a preheated humidified chamber for 1 h at 4 °C. The primary antibodies for eIF4E, eIF4A1, and TSG101 were added, and the slides were incubated overnight at 4 °C. The slides were then washed with buffer and subsequently incubated with the PLA probes and amplification polymerase solution. Fluorescence images were acquired with a confocal laser scanning microscope (LSM 510; Zeiss, Oberkochen, Germany).^[^ ^67^, ^68^ ^]^

### SUnSET Assay

Macrophage (THP1‐derived), endothelial (RF24), mesothelial (Met‐5A, LP3), and fibroblast (GM07213) cells were incubated with EVs (50 µg mL^−1^) isolated from ovarian cancer cells. After 24 h, cells were incubated with puromycin at different time points. Cells were lysed using 1x radio‐immunoprecipitation assay (RIPA) buffer, lysates were run on an SDS‐PAGE gel and transferred to a nitrocellulose membrane for western blotting with antipuromycin antibody. Puromycin staining in lanes was measured using a chemiluminescence kit.

For flow cytometry, THP1‐derived macrophages were incubated with EVs and treated with 100 µg mL^−1^ cycloheximide (CHX) for 1 h followed by incubation with puromycin. Cells were washed with PBS to remove residual puromycin, followed by staining of cells using Alexa fluor‐488 antipuromycin antibody.

### Polysome Fractionation by Sucrose Gradients

Polysome fractionation was performed following a previously published protocol.^[^ ^69^, ^70^ ^]^ In brief, THP1‐derived macrophages were incubated with EVs as described above, and total lysate was prepared. Approximately 10–15 mL of lysate was layered over 20–60% cold sucrose gradients. Gradients were centrifuged in a Beckman SW28 rotor for 2 h at 4 °C. After centrifugation, 12 equal‐sized fractions were collected using a polysome fractionator. For western blotting, fractions were precipitated and mixed with SDS sample buffer. For qPCR, total RNA was isolated from the fractions by mixing with phenol:chloroform:isoamyl alcohol (Sigma‐Aldrich, Cat#15593049), and then RNA was analyzed by qRT‐PCR.

The following primers were used‐

HMGCR‐F:5′‐ACGTGAACCTATGCTGGTC‐3′

HMGCR‐R:5′‐GGTATCTGTTTCAGCCACTAA‐3′

HMGCS1‐F:5′‐AAGTCACACAAGATGCTACA‐3′

HMGCS1‐R:5′‐TCAGCGAAGACATCTGGTG‐3′

### In Vivo Extracellular Vesicle Treatment

*Rab27a^−/−^* ID8 cells were injected orthotopically into the ovary of C57BL/6 mice. Seven days postinjection, EVs were isolated by ultracentrifugation from culture media of ID8 *Trp53^−/−^;Brca2^−/−^* cells, ID8 *Trp53^−/−^;Brca2^−/−^* eIF4E KD, and ID8 *Trp53^−/−^;Brca2^−/−^* eIF4E overexpressed cells. 20 µg EVs were injected into each mouse via the tail vein thrice a week until the control mice became moribund.

### In Vivo Macrophage Treatment

For adoptive transfer, *Csf1r^−/–^* mice (3–5/group) were injected intraperitoneally with ID8 *Trp53^−/−^;Brca2^−/–^* cells. After 10 d, tumor bearing mice were treated with immortalized peritoneal macrophages (PMJ2‐R) twice a week. After 7 weeks, mice were sacrificed, and tumor weight and ascites volume were recorded.

### Isolation and Culturing of Peritoneal Macrophages

Peritoneal macrophages were isolated from black mice by peritoneal lavage as described previously.^[^ ^71^ ^]^ Briefly, cold PBS containing 1% FBS was injected into the peritoneal cavity and extracted after gentle agitation. The peritoneal cell suspension was centrifuged, and cells were resuspended in complete RPMI‐1640 supplemented with 10% FBS and 1% Penicillin‐Streptomycin Solution. Cells were incubated at 37 °C for 2 h to allow macrophages to adhere to the plate. Floating cells were removed by two subsequent washes with PBS. For cell sorting, peritoneal macrophages were stained with CD45 and F4/80 antibody at 4 °C for 1 h in the dark. Samples were then washed and resuspended in FACS running buffer.

### Quantitative Real Time‐PCR (qRT‐PCR)

Total RNA was isolated from the cells using the RNeasy Mini Kit (Qiagen, Valencia, CA, USA), and first‐strand cDNA was transcribed using iScript reverse transcription supermix (Biorad, Hercules, CA, USA, Cat# 1708890). qRT‐PCR was performed using Cycle fluorescent xpression (CFX)C Connect Real‐Time PCR systems (Biorad, Hercules, CA, USA) and iTaqTM Universal SYBR Premix (Biorad, Hercules, CA, USA, Cat# 1725120).

### Stable Cell Line Construction

eIF4E/eIF4A1 overexpressed stable cell lines were developed using a lentivirus vector from Genecopoeia, following the manufacturer's instructions. The stable cell lines were selected by adding puromycin to the culture medium. Lentiviral knockdown cell lines were generated as previously described.^[^ ^72^ ^]^ Briefly, 293T cells were cotransfected with these plasmids: psPAX2, pMD2.G, and pLKO.1 containing shRNA sequence targeting eIF4A1, eIF4E and HMGCR.

sheIF4E ‐5′‐CGATTGATCTCTAAGTTTGAT‐3′; 5′‐CCGAAGATAGTGATTGGTTAT‐3′

sheIF4A1–5′‐GATCTATGACATATTCCAGAA‐3′; 5′‐CGAGAGTAACTGGAACGAGAT‐3′

shHMGCR‐5′‐CCCACAAATGAAGACTTATAT‐3′; 5′‐GAACTTTGCAATCTAAGTTTA‐3′

Two days following cotransfection, competent lentivirus particles were collected. ID8 *Trp53^−/−^;Brca2^−/−^* cells were infected with sheIF4E and sheIF4A1 lentivirus for 48 h. PMJ2‐R cells were infected with shHMGCR lentivirus. Stable knockdown cell lines were selected with puromycin in the cell culture medium.

### CRISPR/CAS9 Knockdown of Genes

*Rab27a* gene knockout ID8 cells were engineered by Synthego (CA, USA) using CRISPR/CAS9 technology. The following guide sequence was used:

RAB27a‐ 5′‐CCUGCAGUUAUGGGACACGG‐3′

### Transcription Factor Array

THP1‐derived macrophages were treated with 10 µm simvastatin for 24 h, and nuclear protein was extracted from treated and untreated cells. The activity of 96 transcription factors in protein extract was measured using the TF Activation Profiling Plate Array II (Signosis) according to manufacturer's instructions, Relevant TFs were selected by the fold change (>2‐fold) between simvastatin treated and untreated THP1‐derived macrophages.

### Luciferase Reporter Assay

Plasmid vector containing the promoter of PD‐L1 was purchased from GeneCopoeia (Cat# HPRM40139‐PG02) and transfected into cells by Lipofectamine‐2000 reagent. The cells were then transfected with XBP1 siRNA or treated with simvastatin for 24 h. Culture medium was then collected and centrifuged at 2000 × *g* for 10 min at 4 °C and 10 µL samples of supernatant were transferred into white‐walled 96‐well plates in triplicate. Luciferase intensity in each well was immediately measured, using a luminometer, as described in the Secrete‐Pair Gaussia Luciferase Assay Kit (GeneCopoeia, Cat# LF061).

### ChIP Assay

The CiiDER tool was used to determine if the PD‐L1 promoter contains a binding site for the transcription factor XBP1. THP1‐derived macrophages were treated with 10 µM simvastatin for 24 h. The ChIP assay was performed using SimpleChIP Enzymatic Chromatin IP Kit (Cell Signaling Technology, Cat# 9003) by adding XBP1 antibody to both samples. Real‐time PCR was used to analyze the amplification of the region when XBP1 binds to the PD‐L1 promoter. ChIP‐PCR primers:

PD‐L1‐F:5′‐ACACACACACACACACCTAC‐3′

PD‐L1‐ R:5′‐CCCCTTCAAGGTGACTGAACAT‐3′

### Cytokine Measurement

Peritoneal macrophages from C57BL/6 naïve mice were treated with EVs (50 µg mL^−1^) derived from the supernatants of the ID8 *Trp53^−/−^;Brca2^−/−^* and eIF4E overexpressed ID8 *Trp53^−/−^;Brca2^−/−^* After incubation, supernatant was isolated from cultured macrophages, centrifuged at 6000 rpm for 5 min to remove cellular debris, and transferred to a new Eppendorf tube. The Cytokine array was performed using the Proteome Profile Mouse Cytokine Array Panel A (R&D Systems, Minneapolis, MN, Cat# ARY006) according to the manufacturer's instructions.

### Mouse T Cell Isolation

Spleen was isolated from C57BL/6 mice, and CD8 T cells were isolated using MojoSort Mouse CD8 T Cell Isolation Kit (Biolegend Cat# 480 008). The cells were stimulated with Dynabeads Mouse T‐Activator CD3/CD8 kit (Invitrogen, Cat# 11456D). T cells were cocultured with macrophages isolated from a separate C57BL/6 mouse that had been pretreated with EVs (50 µg mL^−1^) at a 15:1 ratio. Macrophages were washed before T cell addition to remove residual EVs and recounted. Following culture, cells were surface stained with CD8/BV605 for 30 min. Cells were then fixed and permeabilized using fixation/permeabilization solution kit (BD Bioscience, Cat# 554714) before overnight intracellular staining with IFN‐γ.

### Flow Cytometry

The single‐cell suspension was first incubated with FC block, then live–dead staining was performed, and the cells were washed with stain buffer. Cell surface antigens were stained by coincubation with antibodies for 30 min on ice, followed by washing with stain buffer. Staining of intracellular antigen was performed with a BD Pharmingen kit according to the manufacturer's protocol. Flow cytometry was performed on a Fortessa X20 and Cytek Aurora, and data were analyzed by FlowJo software.

### Stable Isotope Labeling by/with Amino Acids in Cell culture (SILAC) Based Mass Spectrometry

SILAC RPMI (Pierce Biotechnology) was supplemented with 10% dialyzed fetal bovine serum (Thermo Scientific, Waltham, MA, USA, Cat# A3382001), 1% streptomycin/penicillin. The heavy medium was supplemented with ^13^C_6_ L‐arginine (Thermo Scientific, Waltham, MA, USA, Cat# 88210) and ^13^C_6_^15^N_2_‐L‐lysine (Thermo Scientific, Waltham, MA, USA, Cat# 88 209). The light medium was supplemented with normal L‐arginine and L‐lysine. For SILAC experiments, THP1‐derived macrophages were grown in parallel in either light or heavy media for 5 d, with media replacement every 24 h. Cells grown in light medium were incubated with 50 µg mL^−1^ control EVs, and cells with heavy medium were incubated with 50 µg mL^−1^ eIF4E‐EVs for 24 h.

The SILAC‐labeled cell pellets were lysed and analyzed on a Thermo Scientific Orbitrap Fusion Lumos MS via 3 technical replicate injections using a DDA as described earlier.

Peptides and proteins were both filtered for 0.01 false discovery rate (FDR), proteins a minimum two unique peptides, precursor abundance based on area, unique and razor peptides used for quantification, normalized on total peptide amount. Quantified proteins with ≥ 2 and ≤ 0.5‐fold change were selected and clustered by biological functions, pathway, and network analysis using IPA software (www.ingenuity.com) for bioinformatics analysis. A heat map for differentially expressed proteins was generated using heatmapper (http://www.heatmapper.ca/).

### Metabolism Assays—Seahorse XF Cell Mito Stress Test

An XF96e Analyzer (Agilent Technologies, Santa Clara, CA) was used to measure bioenergetic function in isolated peritoneal macrophages and THP1‐derived macrophages as described earlier.^[^ ^31^ ^]^ In brief, cells were stimulated with control and eIF4E‐EVs (50 µg mL^−1^) overnight. For all bioenergetic measurements, the culture media were changed 1 h before the assay run to unbuffered Agilent Seahorse XF RPMI Medium supplemented with D‐Glucose, Pyruvate, and glutamine. Three basal OCR measurements were recorded before injection of oligomycin. After recording the oligomycin‐sensitive OCR, carbonyl cyanide‐4‐(trifluoromethoxy) phenylhydrazone (FCCP)‐sensitive rates were recorded. Finally, antimycin A/rotenone was injected to inhibit electron flow through the electron transport system.

### Seahorse Glycolytic Stress Assay

Cells were stimulated with control and eIF4E‐EVs (50 µg mL^−1^) overnight. The culture media was changed 1 h before the assay run to unbuffered Agilent Seahorse XF RPMI Medium supplemented with glutamine. Three basal ECAR measurements were recorded, followed by an injection of a saturating level of glucose, measuring a glucose‐induced response. This was followed by Oligomycin A, an ATP synthase inhibitor, which inhibits mitochondrial ATP production and shifts the energy production to glycolysis, revealing the cellular maximum glycolytic capacity. Finally, 2‐deoxyglucose, a glucose analog, was injected to inhibit glucose binding to hexokinase, thus confirming extracellular rates to be a product of glycolysis.

### In Vitro Metabolism Assays

Peritoneal macrophages and THP1‐derived macrophages were stimulated with control and eIF4E‐EVs (50 µg mL^−1^) overnight. Cells were then washed and incubated in glucose free RPMI for 30 min before 100–200 µg mL^−1^ 2‐NBDG (Caymen Cat# 600470), in glucose‐free medium was added. Cells were incubated with 2‐NBDG for 2 h before washing. 2‐NBDG taken up by cells was detected with a fluorescence plate reader at excitation and emission wavelengths of 485 and 535 nm, respectively.

### Cholesterol Content Measurement

Peritoneal macrophages and THP1‐derived macrophages were stimulated with control and eIF4E‐EVs overnight. After incubation, lipid was extracted and total cellular cholesterol content was measured in macrophages using Amplex Red Cholesterol Assay kit (Invitrogen, Cat# A12216), according to the manufacturer's instructions. EVs treated macrophages were also cocultured with tumor cells. After incubation, tumor cells were washed, and cholesterol content was measured in tumor cells. For the detection assay, cells were stained with Filipin III and then analyzed by flow cytometry.

### Metabolite Extraction

Steady state isotope tracing analysis using mass spectrometry was performed as reported earlier.^[^ ^73^, ^74^, ^75^ ^]^ Briefly, THP1‐derived macrophages were seeded in six‐well plates overnight and replaced with medium containing U‐^13^C_6_ glucose (Cambridge Isotope Lab, Inc., Cat# CLM‐1396–0) or U‐^13^C_5_ glutamine (Cambridge Isotope Lab, Inc., Cat# CLM‐1822‐H). Cells were treated with control EVs and eIF4E‐EVs (50 µg mL^−1^) for 48 and 96 h, and metabolites were extracted.

LC‐MS/MS for Untargeted Metabolomics: Dried extracts were reconstituted in methanol/water (50:50), sonicated, and filtered. 5 µL for the sample was injected for untargeted analysis on Kinetex F5 column (2.1×150 mm^2^, 2.6 µm) in both positive and negative ion modes. The column was kept at 30 °C and the flow rate was 0.2 mL min^−1^. The ScieXOS software was used for peak integration and analysis.

GC‐MS for Cholesterol: Dried cholesterol samples were derivatized with trimethylsilyl (TMS) and incubated at 37 °C for 30 min. Samples were analyzed using Agilent 7890 GC equipped with a 30‐m HP‐5MSUI capillary 1246 column connected to an Agilent 5977B MS in scan mode. 1–2 µL of sample was injected at 270 °C with helium as the carrier gas at 1 mL min^−1^ flow. The temperature gradient was maintained at 260 °C for 3 min, raised to 280 °C for 15 min, increased to 325 °C for 15 min, and held for 15 min. The quadrupole was operated at 150 °C.

GC‐MS for Polar Metabolites: Dried samples were derivatized using methoxyamine hydrochloride (MOX, FisherScientific, PI45950), followed by incubation at 45 °C for 30 min with constant shaking. Next, 30 µL of N‐tert‐butyldimethylsilyl‐N‐methyltrifluoroacetamide (MBTSTFA)+1% tert‐butyldimethylsilyl (TBDMCS) (Sigma‐Aldrich, M‐108‐12435×1ML) was added, and the samples were incubated at 45 °C for 1 h. 1–2 µL of the derivatized sample was injected at 270 °C with helium as the carrier gas at 1 mL min^−1^ flow. The temperature gradient method was set up as 100 °C for 1 min, raised at 3.5 °C min^−1^ to 255 °C, increased to 320 °C at 15 °C min^−1^, and held for 3 min (total time 52.6 min). MS detector was operated in scan mode (70–600 m/z). A calibration curve was run for polar metabolite standards (0–10 nmoles).

### Quantification and Statistical Analysis

Statistical analysis was done using GraphPad Prism 9.5.0 and presented as mean values ± SEM. Student's *t*‐test was performed to calculate *p*‐values to compare two groups. For more than two groups, one‐way ANOVA and post hoc Dunnett's multiple comparison tests were used to compare data from the control and each test group. Throughout all figures: ∗*p* < 0.05, ∗∗*p* < 0.01, ∗∗∗*p* < 0.001, and ∗∗∗∗*p* < 0.0001. Significance was defined at *p* < 0.05. Overall survivals were assessed with the Kaplan‐Meier method and compared with the log‐rank test.

## Conflict of Interest

The authors declare no conflict of interest.

## Author Contributions

S.M., P.C.R., and S.P. initiated the study and designed the experiments. S.M. performed most of the experiments, statistical analysis, prepared figures and drafted the paper. M.N., O.A., N.M., A.S. helped with LC‐ MS and GC‐MS experiments and analysis. S.‐W.T. performed all the bioinformatics and computational analysis for this study. I.P.K., S.K. J.G., P.G., M.S., A.G., and C.D. assisted with animal experiments and in vitro experiments. P.C.R. provided scientific feedback and assisted with translational experiments. D.N. provided scientific feedback and assisted in designing and executing LC‐MS and GC‐MS experiments. S.P. established collaborations and allocated funding for the work. S.M. prepared the manuscript with S.P. All authors have read and approved the article.

## Supporting information



Supporting Information

Supplemental Table 1

Supplemental Table 2

## Data Availability

The data that support the findings of this study are available from the corresponding author upon reasonable request.
