# Supplementary material for: eIF4E Enriched Extracellular Vesicles Induce Immunosuppressive Macrophages through HMGCR‐Mediated Metabolic Rewiring
Source: Adv Sci (Weinh). 2025 Aug 18;12(42):e06307. doi: 10.1002/advs.202506307 (PMC12622447; doi:10.1002/advs.202506307)
Supplement: Supplementary file 1 — Supporting Information [file ADVS-12-e06307-s001.docx]

**eIF4E Enriched Extracellular Vesicles Induce Immunosuppressive Macrophages Through HMGCR-Mediated Metabolic Rewiring**

**Authors-** Sonam Mittal^1^, Minal Nenwani^2,3^, Ishaque Pulikkal Kadamberi^1^, Sudhir Kumar^1,4^, Olamide Animasahun^3,5^, Jasmine George^1^, Shirng-Wern Tsaih^1^, Prachi Gupta^1,6^, Mona Singh^1^, Anjali Geethadevi^1^, Chandrima Dey^1^, Noah Meurs^2,3^, Ajay Shankaran^3,5^, Pradeep Chaluvally Raghavan^1,7,8^, Deepak Nagrath^2,3,5,9^, Sunila Pradeep^*1,7,8^


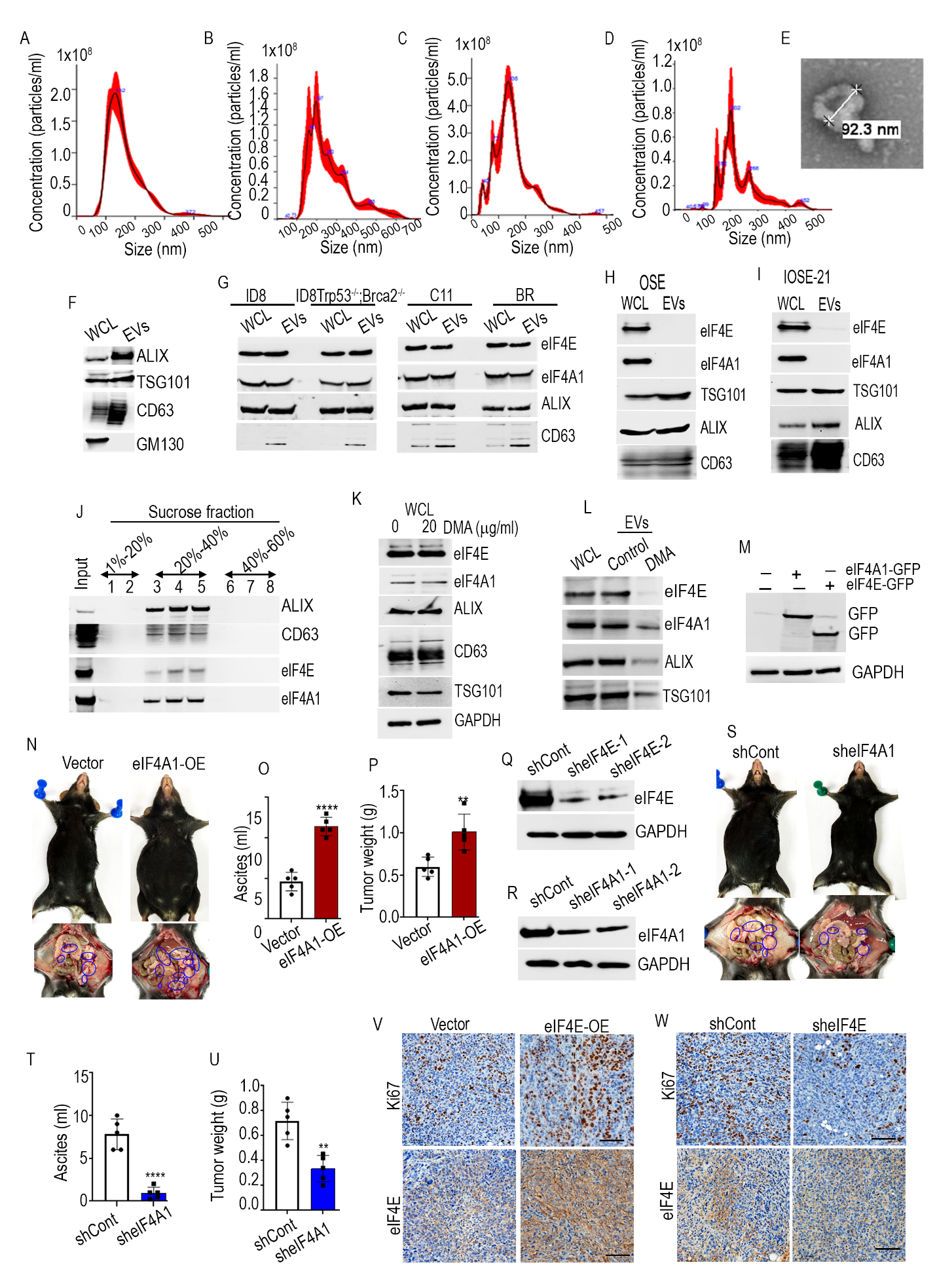


**Sup Fig. 1.** Characterization of purified EVs from A) OVCAR5, B) Hey A8, C) OVCAR8, and D) ID8 *Trp53^-/-^;Brca2^-/-^* cells using nanoparticle tracking (NTA). E) Representative TEM image of purified EVs from OVCAR5 cells. F) Western blotting of EVs positive (TSG101, CD63, ALIX) and negative markers, GM130. G-I) Western blot analysis of eIF4E and eIF4A1 proteins on whole cell lysates and isolated EVs. J) Western blotting using sucrose gradient fractions. K,L) Western blotting of DMA-treated OVCAR8 cells. M) Western blotting of GFP-tagged eIF4A1 and eIF4E was overexpressed in ID8 *Trp53^-/-^;Brca2^-/-^* cells. N) Representative image of tumor bearing mice from vector control and eIF4A1 overexpressed groups (n=5). O) Bar graph representing ascites volume and P) tumor weight of mice from vector control and eIF4A1 overexpressed groups. Q,R) Efficiency of eIF4E and eIF4A1 KD in ID8 Tr*p53^-/-^;Brca2^-/-^* cells using shRNA (sheIF4E and sheIF4A1, respectively). S) Representative image of tumor-bearing mice from the sh-control and sheIF4A1 groups (n=5). T) Bar graph representing ascites volume and U) tumor weight of mice from vector control and eIF4A1 overexpressed groups. V,W) IHC analysis of tumor tissues selected from each group. Scale bar 100µm. The data are shown as mean ± SEM. **p<0.01, ****p<0.0001 compared to control.


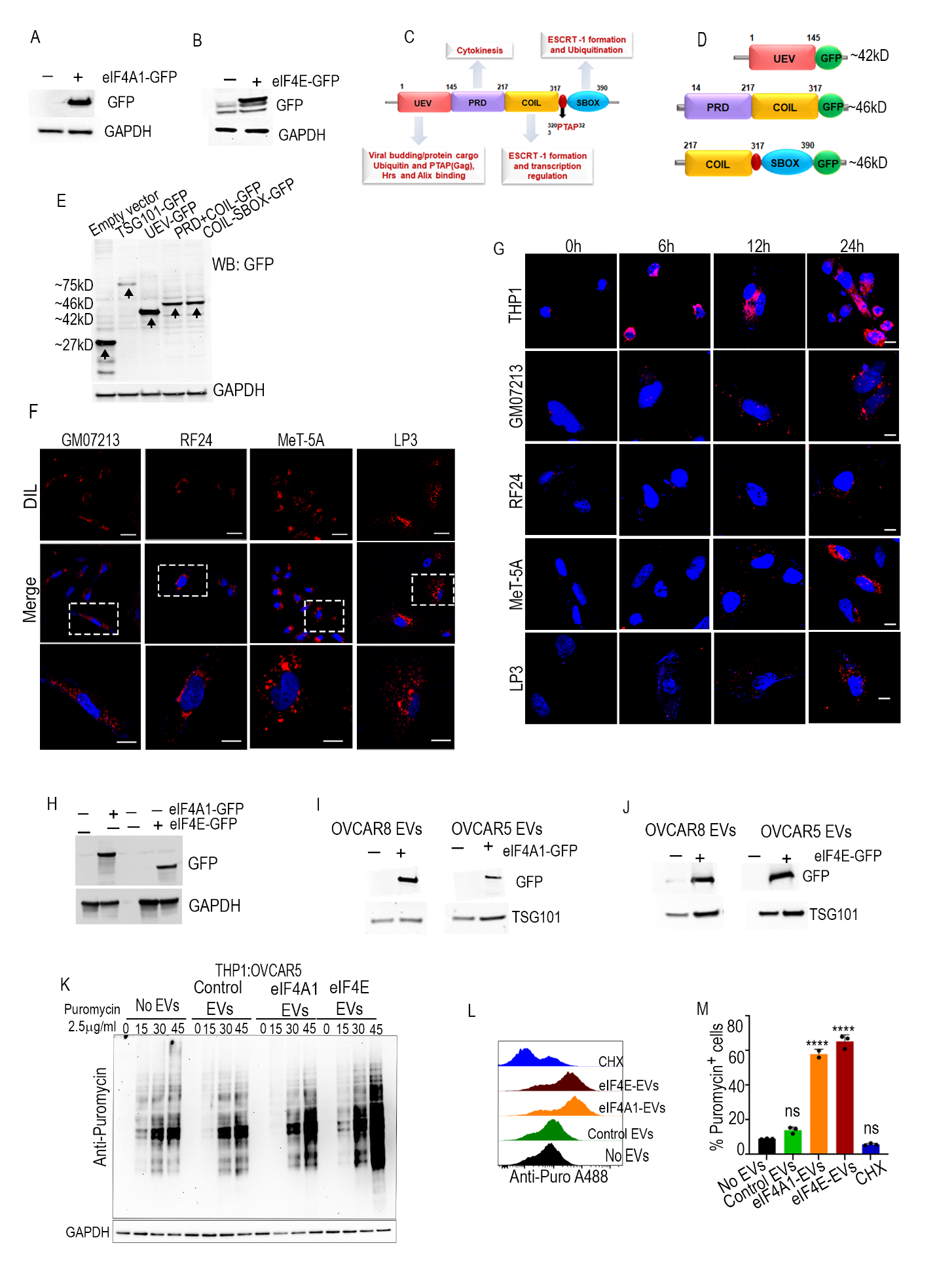


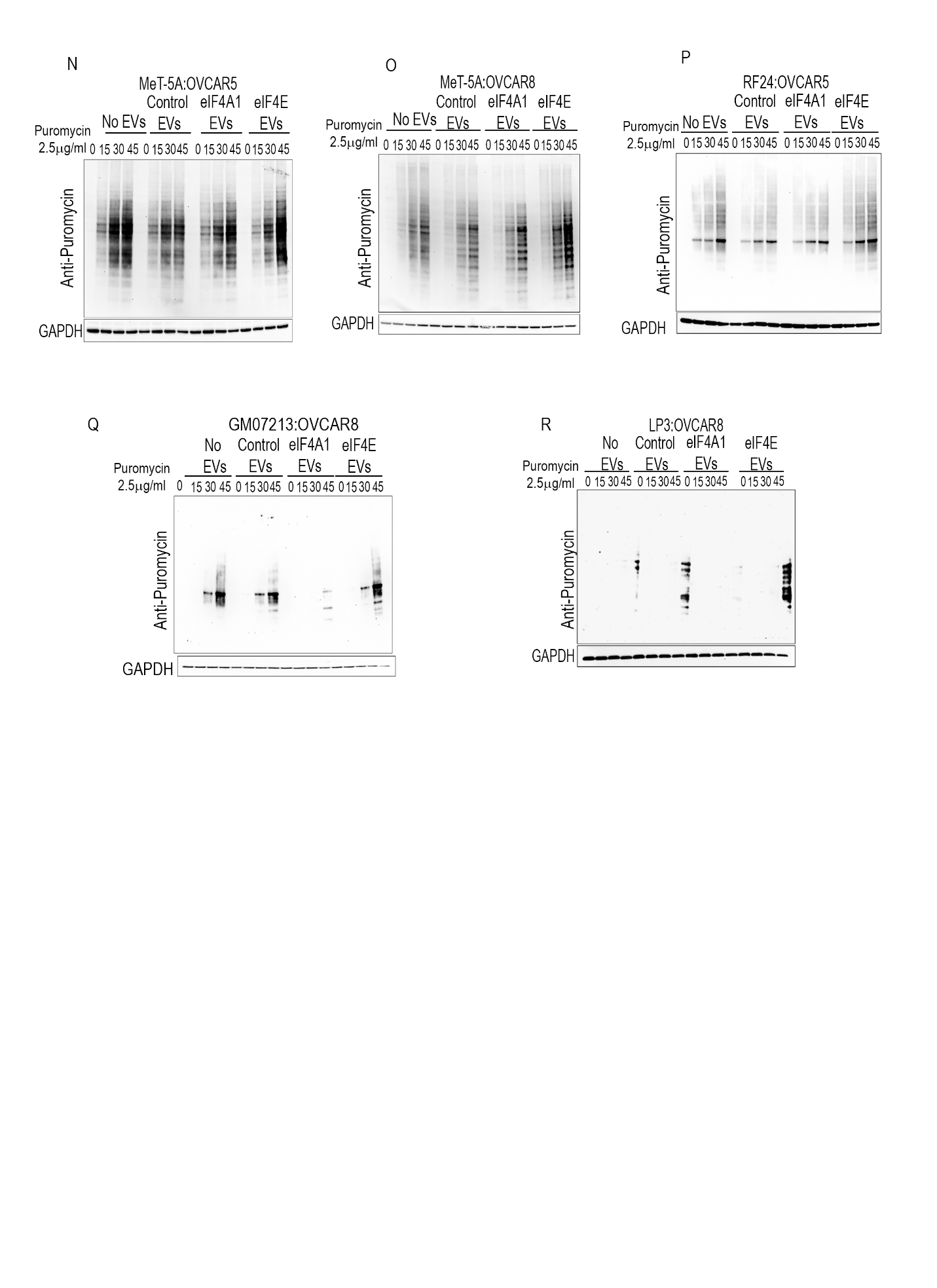


**Sup Fig. 2.** A,B) Western blotting of GFP-tagged eIF4A1 and eIF4E overexpressed in OVCAR8 cells. Schematic description of C) full-length TSG101 and D) truncated TSG101. E) Western blotting showing the expression of full-length and truncated TSG101 proteins with GFP tag at the C-terminal in OVCAR8 cells. F) The uptake of EVs by the indicated cells. Scale bar 50 µm and 20 µm. G) DIL-labeled EVs expression in different cell lines for various time points. Scale bar 50 µm. H) Western blotting of GFP-tagged eIF4A1 and eIF4E overexpressed in OVCAR5 cells. I,J) Western blotting of EV protein isolated from WT and eIF4A1-GFP/eIF4E-GFP overexpressed ovarian cancer cells. K) SUnSET measurements of protein synthesis by western blotting in THP1-derived macrophages after treatment with EVs isolated from OVCAR5 cells. L,M) FACS of the puromycin-labeled THP1-derived macrophage using the anti-puromycin antibody tagged with Alexa Fluor-488. N-R) SUnSET measurements of protein synthesis by western blotting after treatment with EVs isolated from ovarian cancer cells. Error bars indicate mean ± SEM, ****p<0.0001 (one-way ANOVA).


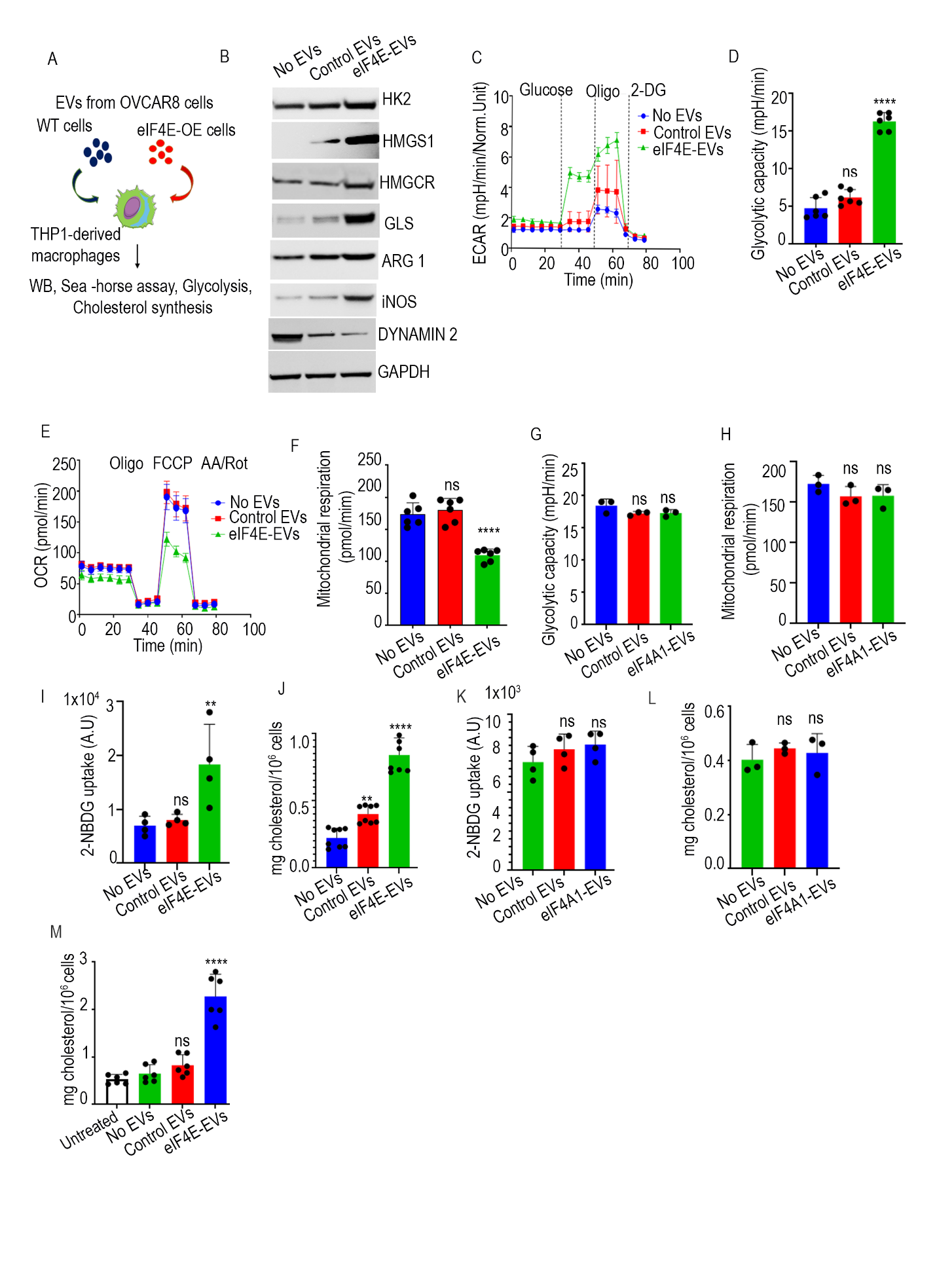
**Sup Fig. 3.** A) Workflow representing validation of SILAC. B) Representative western blots showing expression of HK2, HMGCS1, HMGCR, GLS, ARG 1, iNOS, and DYNAMIN 2, in macrophages treated with EVs. C,D) Seahorse glycolysis stress test with sequential addition of glucose, oligomycin, and 2-DG in macrophages pre-treated with eIF4E-EVs. E,F) OCR measurement in eIF4E-EVs stimulated macrophages (n=3). G) Seahorse glycolysis stress test with sequential addition of glucose, oligomycin, and 2-DG in macrophages pre-treated with eIF4A-EVs (n=3). H) OCR measurement in eIF4A1-EVs stimulated macrophages. Macrophages (n=3). I) Glucose uptake assay with 2-NBDG in macrophages stimulated with EVs (n=3). J) Cholesterol synthesis in macrophages stimulated with eIF4E-EVs by Amplex cholesterol assay. K) Glucose uptake assay with 2-NBDG in macrophages stimulated with eIF4A1-EVs (n=3). L) Cholesterol synthesis in macrophages stimulated with eIF4A1-EVs by Amplex cholesterol assay (n=3). M) Cholesterol measurement in OVCAR8 cells co-cultured with THP1-derived macrophages treated with EVs (n=3). Error bars indicate mean ± SEM, *p<0.05, **p<0.01 ****p<0.0001 (one-way ANOVA).


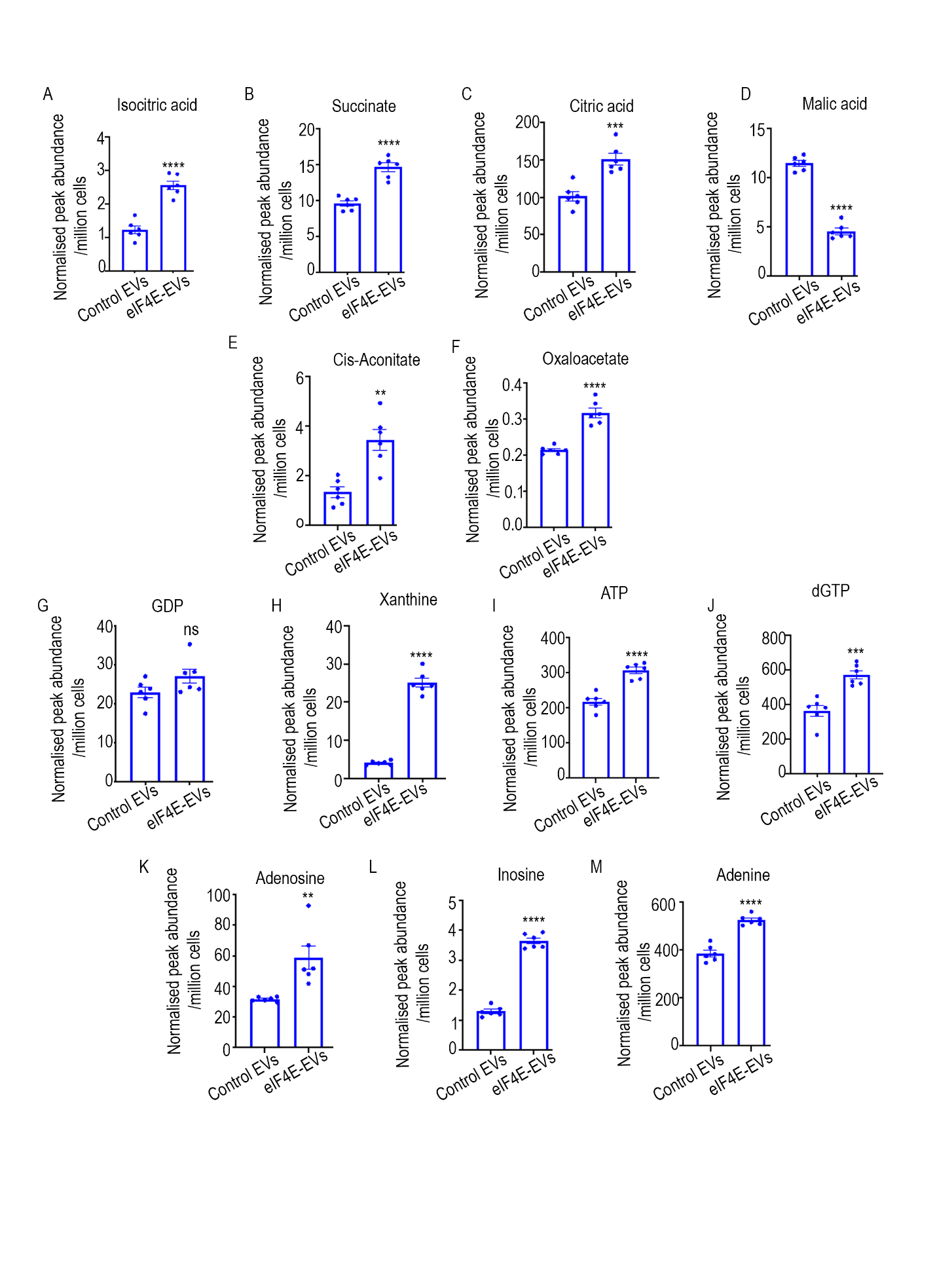
**Sup Fig. 4**. A-M) Relative metabolite levels (peak intensity normalized by internal standard and cell number in macrophages treated with control and eIF4E-EVs (n=6). **p<0.01, ***p<0.001, ****p<0.0001, (Student’s t-test). ns, non-significant.


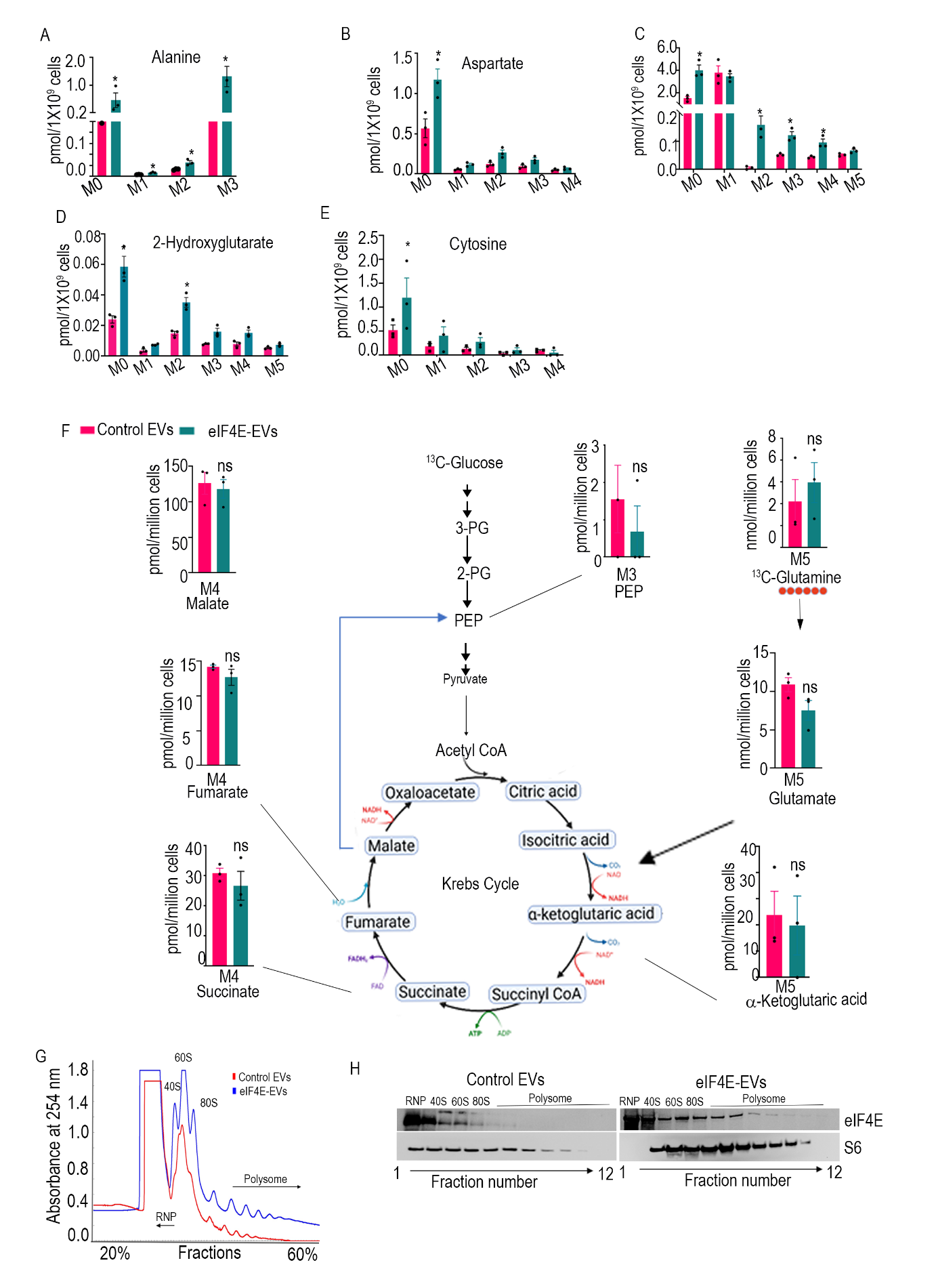


**Sup Fig. 5.** A-E) Fractional contribution of uniformly labelled ^13^C glucose in control and eIF4E-EV treated macrophages (n=3). F) Fractional contribution of uniformly labelled ^13^C glutamine in central carbon metabolites in control and eIF4E-EV treated macrophages (n=3). G) Plot of the absorbance profile of fractions obtained through sucrose gradients to isolate polysomes. H) Western blot analysis of the protein fractions isolated from G) was performed using the antibodies indicated. Error bars indicate mean ± SEM, *p<0.05. ns, non-significant.


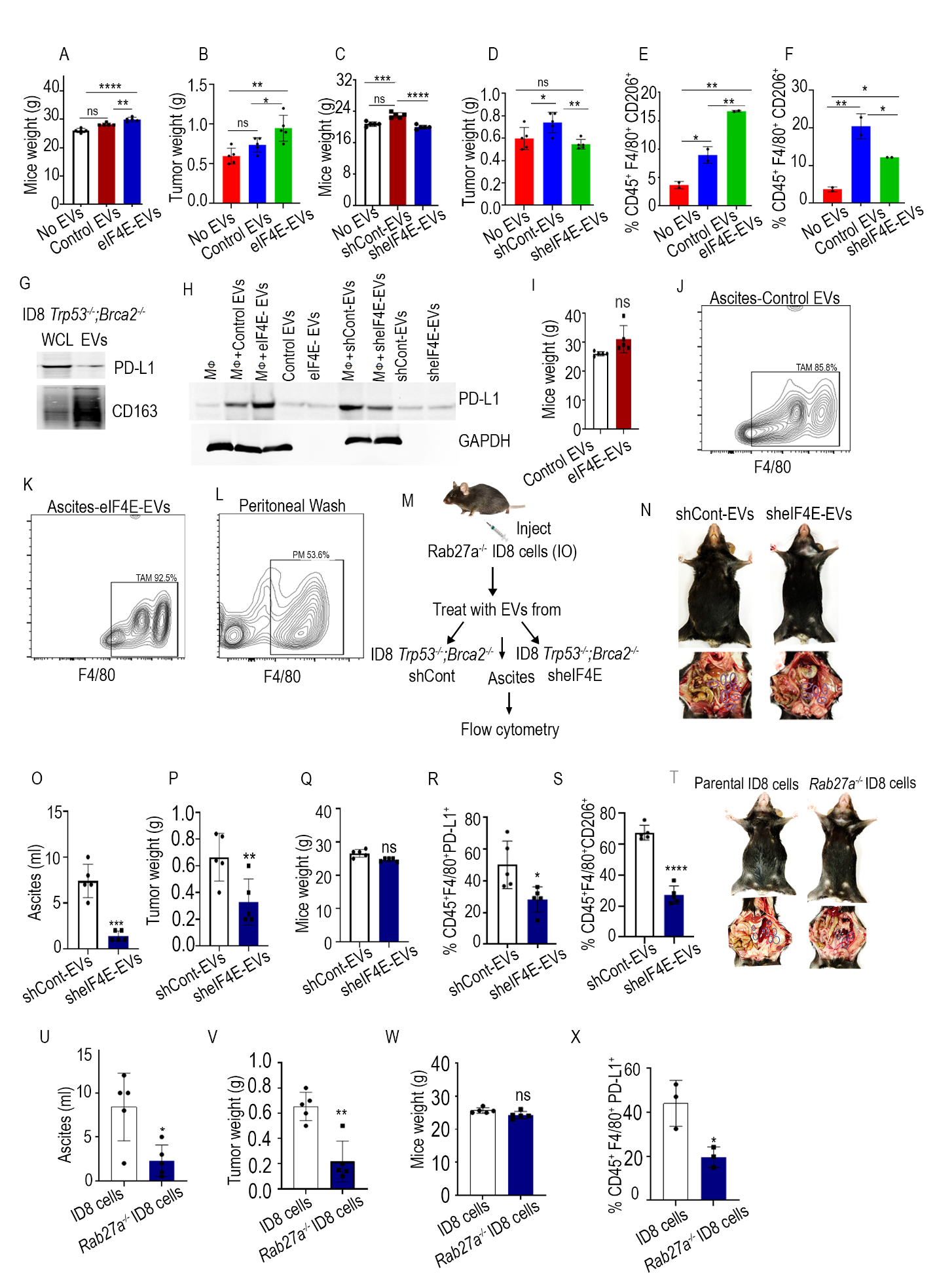


**Sup Fig. 6.** The bar graph indicates A) average mice weight and B) average tumor weight from control EVs and eIF4E-EVs groups. The bar graph indicates C) average mice weight and D) average tumor weight from shCont-EVs and sheIF4E-EVs groups. E,F) The bar graph representing CD206 expression on macrophages in ascites samples (n=3). G) Representative western blot analysis of PD-L1 expression in EVs from ID8 *Trp53^-/-^;Brca2^-/-^* cells. H) Representative western blot analysis of PD-L1 expression in macrophages, macrophages stimulated with EVs isolated from eIF4E OE/KD ID8 *Trp53^-/-^;Brca2^-/-^* cells (20 μg), and ID8 *Trp53^-/-^;Brca2^-/-^* EVs alone (20 μg). I) Bar graph indicates the average mice weight. J-L) Total macrophage infiltration from peritoneal wash and ascites from tumor bearing mice. M) Schematic representation for experimental design N-S). N) Peritoneal cavity of the mice showing ascites accumulation and tumor locations (blue circles) (n=5). O) Bar graph representing average ascites volume, P) tumor weight, and Q) mice weight. R) PD-L1 expression S) CD206 expression on macrophages in ascites samples (n=5). T) Peritoneal cavity of the mice showing ascites accumulation and tumor locations (blue circles) (n=5). U) Bar graph representing average ascites volume. V) tumor weight, W) mice weight, and X) PD-L1 expression. Error bar represents ±, SEM, *p<0.05, **p<0.01, ***p<0.001, ****p<0.0001 (Student’s t-test). ns, non-significant.


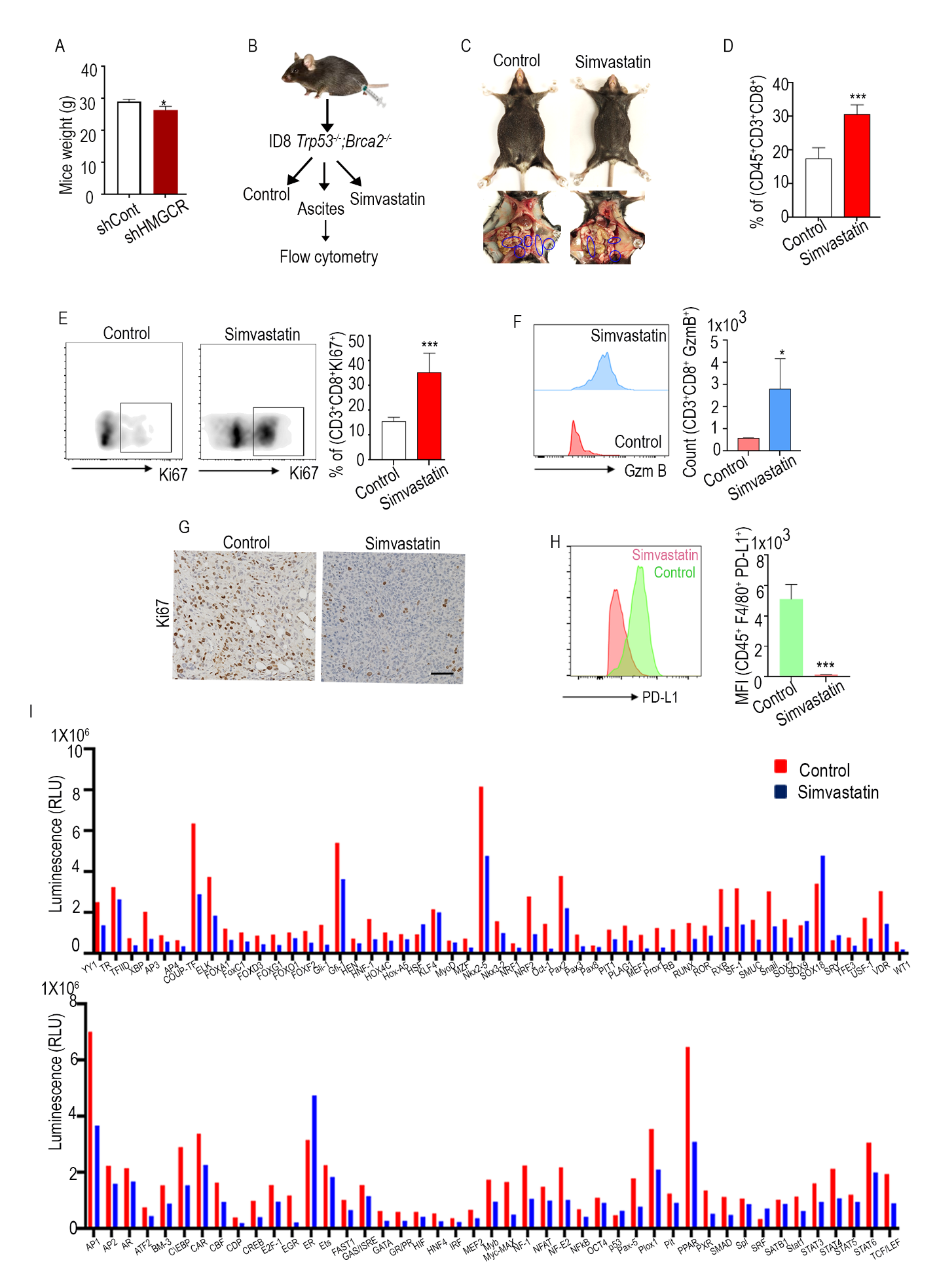


**Sup Fig. 7.** A) Bar graphs indicate mice weight. B) Schematic representation of the simvastatin treatment (10mg/kg). C) Peritoneal cavity of mice showing ascites accumulation and tumor nodules in mice treated with vehicle control and simvastatin (n=7). D) Flow cytometric quantification of CD8^+^ cells among CD45^+^ and CD3^+^ cells in ascites. E,F) Quantification of Ki67^+^ cells and GzmB^+^ cells among CD8^+^ T cells in ascites. G) IHC analysis of tumor tissues. H) Flow cytometry analysis of PD-L1 expression in peritoneal macrophages (n=3). I) The Bar graph gives the results of a transcription factor array in macrophages treated with simvastatin. Error bar represents ±, SEM, *p<0.05, ***p<0.001 (Student’s t-test).


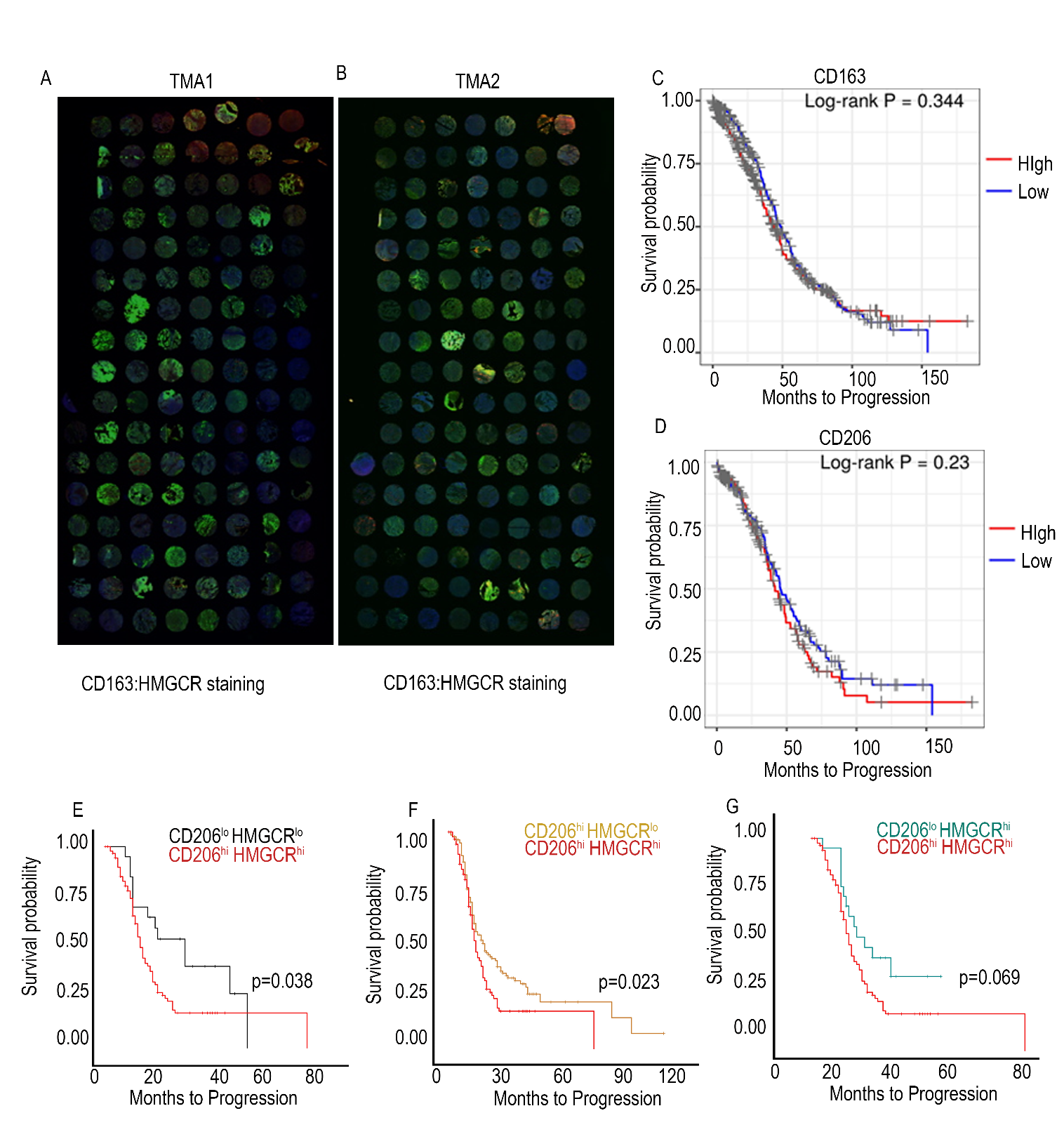


**Sup Fig. 8.** A,B) Co-Immunofluorescence staining for HMGCR and CD163 in the whole slide of human ovarian cancer tissue microarray of 252 cores. C) Kaplan-Meier survival curve of high CD163 vs low CD163 expression patients. D) High CD206 vs low CD206 expression patients. E) High HMGCR and high CD206 expression (n=91) vs. low HMGCR and low CD206 expression (n=20). F) High HMGCR and high CD206 expression (n=91) vs. low HMGCR and high CD206 expression (n=141). G) High HMGCR and high CD163 expression (n=91) vs. high HMGCR and low CD206 expression (n=22). Error bar represents ±, SEM, *p<0.05, (Student’s t-test)

**Antibodies and reagents**

| **Reagent or resource** | | **Source** | **Identifier** |
| --- | --- | --- | --- |
| **Antibodies** | |  |  |
| TSG101 | | Proteintech | Cat# 14497-I-AP; RRID:AB_2208090 |
| CD63 | | Proteintech | Cat# 25682-I-AP; RRID:AB_2208090 |
| Alix | | Santa Cruz Biotechnology | Cat# sc-53540; RRID:AB_673819 |
| TSG101 | | Thermo Fisher Scientific | Cat# MA1-23296; RRID:AB_2208088 |
| Alexa Fluor 568 goat anti-rabbit | | Life Technologies | Cat# F2765,  RRID: AB_10562896 |
| Alexa Fluor goat anti-mouse 488 | | Life Technologies | Cat# F2761, RRID: AB_1500661 |
| Anti-rabbit IgG, HRP-linked | | Cell Signaling Technology | Cat#: 7074S: RRID:AB_2099233 |
| Anti-mouse IgG, HRP-linked | | Cell Signaling Technology | Cat#: 7076P2: RRID:AB_330924 |
| GFP | | Santa Cruz Biotechnology | Cat# Sc-9996; RRID:AB_627695 |
| Anti-puromycin | | Millipore Sigma | Cat# MABE343, RRID: AB_2566826 |
| Anti-puromycin | | Sigma Aldrich | Cat# MABE343; RRID: AB_2566826 |
| Anti-Puromycin, clone 12D10, Alexa Fluor® 488 Conjugate | | Millipore Sigma | Cat# MABE343-AF488; RRID: AB_2736875 |
| eIF4A1 | | Sigma-Aldrich | Cat#SAB1300839; RRID: AB_10606599 |
| eIF4E | | Sigma-Aldrich | Cat# E5906, RRID: AB_796203 |
| eIF3B | | Proteintech | Cat# 10319-1-AP; RRID: AB_2096732 |
| eIF3I | | Proteintech | Cat# 11287-1-AP; RRID: AB_2097068 |
| eIF3E | | Proteintech | Cat# 11352-1-AP; RRID: AB_2277716 |
| eIF3L | | Proteintech | Cat# 11482-1-AP; RRID: AB_2877768 |
| eIF3A | | Proteintech | Cat# 26178-1-AP; RRID: AB_2880413 |
| eIF5A2 | | Proteintech | Cat# 17069-1-AP; RRID: AB_2262009 |
| eIF4B | | Cell Signaling Technology | Cat# 3592S; RRID: AB_2293388 |
| eIF4G | | Cell Signaling Technology | Cat# 2469S; RRID:AB_2096028 |
| eIF4A | | Cell Signaling Technology | Cat# 2013S; RRID:AB_2097363 |
| eIF4H | | Cell Signaling Technology | Cat# 3469S; RRID:AB_2096038 |
| XBP-1s | | Cell Signaling Technology | Cat# 40435; RRID:AB_2891025 |
| GAPDH | | Cell Signaling Technology | Cat# 5174S; RRID:AB_10622025 |
| HMGCR | | Proteintech | Cat# 13533-1-AP; RRID: AB_2877957 |
| HMGCS1 | | Proteintech | Cat# 17643-1-AP; RRID: AB_ 2248359 |
| HK2 | | Proteintech | Cat# 22029-1-AP; RRID: AB_11182717 |
| Arginase1 | | Proteintech | Cat# 16001-1-AP; RRID: AB_2289842 |
| Dynamin2 | | Proteintech | Cat# 14605-1-AP; RRID AB_2277414 |
| Zombie UV™ Fixable Viability Kit | | Biolegend | Cat#: 423107 |
| Anti-mouse CD4-BV711 | | Biolegend | Cat# 100447; RRID: AB_2564586 |
| Anti-mouse CD8-BV605 | | Biolegend | Cat# 100744; RRID:AB_2562609 |
| Anti-mouse CD45-BV510 | | Biolegend | Cat# 103138; RRID:AB_2563061 |
| Anti-mouse CD3-BUV395 | | BD Horizon | Cat# 563565; RRID:AB_2738278 |
| Anti-mouse PD1-PE | | BD Pharmingen | Cat# 561788; RRID:AB_10895570 |
| Anti-mouse TIM3-BV421 | | BD Biosciences | Cat# 747626; RRID:AB_2744192 |
| Anti-Mouse CD206-BV711 | | Biolegend |  |
| Anti-Mouse CD274-BV605 | | Biolegend | Cat# 124321;RRID:AB_2563635 |
| Anti-Mouse CD206-BV605 | | Biolegend | Cat# 141721;RRID: AB_2562340 |
| Anti-Mouse CD11b-PE | | Biolegend | Cat# 101208 RRID:AB_312791 |
| Anti-Mouse F4/80- Alexa fluor 488 | | Biolegend | Cat#123120; RRID:  AB 893479 |
| **Chemicals, peptides, and recombinant proteins** | |  |  |
| RIPA buffer | | Thermo Fisher Scientific | Cat# 89901 |
| ImmPRESS HRP Horse Anti-Rabbit IgG PLUS Polymer Kit | | Vctorlabs | Cat# MP-7801-15 |
| ImmPRESS HRP Horse Anti-Mouse IgG PLUS Polymer Kit | | Vctorlabs | Cat# MP-7802-15 |
| GFP-TRAP Dynabeads beads | | Chromotek | RRID: AB_2631358 |
| HaltTM Protease & Phosphatase inhibitor cocktail | | Thermo Fisher Scientific | Cat# 78444 |
| Pierce BCA Assay Protein kit | | Thermo Fisher Scientific | Cat# 23225 |
| ECL Prime western blotting detection reagent | | Thermo Fisher Scientific | Cat# 34580 |
| G418 | | Thermo Fisher Scientific | Cat# J63871-AB |
| ACK lysis buffer (RBC lysis buffer) | | Quality Biological | Cat# 118-156-101 |
| FACS buffer | | Tonbo Biosciences | Cat# 4222-L500 |
| Cyto-Fast™ Fix/Perm Buffer buffer | | Biolegend | Cat# 426803 |
| TF Perm/Wash Buffer (5X) | | BD | Cat# 562725 |
| Polybrene | | Sigma | Cat# 107689 |
| Bovine serum albumin (BSA) | | Sigma | Cat# A3059 |
| Opti-MEM(1X) | | Gibco | Cat#31985-070 |
| Dulbecco’s Modified Eagle  Medium (DMEM) | | Thermo Fisher Scientific | Cat# 10569010 |
| ITS | | Thermo Fisher Scientific | Cat# 41400045 |
| Bodipy 493/503 | | Cayman Chemical | Cat# 25892 |
| Simvastatin | | Millipore Sigma | Cat# S6196 |
| Filipin | | Millipore Sigma | Cat# F4767 |
| Fetal bovine serum (FBS) | | Atlanta Biologicals | Cat# H17112 |
| Hanks' balanced salt solution (HBSS) | | Atlanta Biologicals | Cat# 14170120 |
| Antibiotic (Penicillin/Streptomycin | | Thermo Fisher Scientific | Cat# 15140122 |
| Lipofectamine 2000 | | Thermo Fisher Scientific | Cat# 11668027 |
| Vybrant™ DiI Cell-Labeling Solution | | Thermo Fisher Scientific | Cat# V22885 |
| DPBS | | Sigma | Cat# D8662 |
| psPAX2 | | Addgene | Cat# 12260 |
| pMD2.G | | Addgene | Cat# 12259 |
| pLCP-GFP | | Addgene | Cat# 17448 |
| pLCP-GFP-eIF4E | | Genecopia | Cat# EX-Mm20551-Lv182 |
| pLCP-GFP-eIF4A1 | | Genecopia | Cat# EX-Mm20550-Lv182 |
| **Experimental models: Cell lines** | |  |  |
| 293T | | ATCC | Cat# CRL-3216 |
| ID8 | | Sigma | Cat# SCC145 |
| **Experimental models: Organisms/strains** | | | |
| 4-6 weeks old Female C57BL/6 mice | Envigo | | - |
| 4-6 weeks old Female C57BL/6 mice B6.Cg-Csf1r<tm1.2Jwp>/J | Jackson Laboratory | |  |
| **Critical commercial assays** | | | |
| SimpleChIP® Enzymatic Chromatin IP Kit (Magnetic Beads) | | Cell Signaling Technology | Cat# 9003 |
| Luc-Pair Duo-Luciferase HS Assay Kit | | GeneCopoeia | Cat# LF004 |
| Miniprep Kit | | QIAGEN | Cat# 27104 |
| PLA Kit in situ detection reagent | | Sigma Aldrich | Cat# DUO92008 |
| Glucose Uptake CellBased Assay Kit | | Cayman Chemical | Cat# 600470 |
| Glycolysis CellBased Assay Kit | | Cayman Chemical | Cat# 600450 |
| Proteome Profiler Mouse Cytokine Array Kit, Panel A | | R&D Systems | Cat# ARY006 |
| MojoSort™ Mouse CD8 T Cell Isolation Kit | | Biolegend | Cat# 480008 |
| Seahorse XF Glycolysis Stress Test Kit | | Agilent | Cat# 103020-100 |
| Seahorse XF Cell Mito Stress Test Kit | | Agilent | Cat# 103015-100 |
| Dynabeads Mouse T-Activator CD3/CD28 for T Cell Expansion and Activation | | Invitrogen | Cat# 11456D |
| iScript™ cDNA Synthesis Kit | | Bio-Rad | Cat# 1778890 |
| iTaq™ Universal SYBR® Green Supermix | | Bio-Rad | Cat# 1725120 |
| Amplex Red Cholesterol Assay Kit | | Invitrogen | Cat# A12216 |
| Fixation/Permeabilization Solution Kit | | BD Biosciences | Cat# 554714 |
| RNeasy Mini Kit | | QIAGEN | Cat# 74106 |
| **Software and Algorithm** | |  |  |
| GraphPad Prism 9.5 | | GraphPad | https://www.graphpad.com/ |
| FlowJo™ v10.10 | | FlowJo LLC | https://www.flowjo.com/ |
| NTA software 3.1 | | Malvern, Worcestershire,  United Kingdom | https://www.malvernpanalytical.com |
| ImageJ Fiji, v1.54i | | N/A | https://fiji.sc/ |
